# A simple PCR-based quick detection of the economically important oriental fruit fly, *Bactrocera dorsalis* (Hendel) from India

**DOI:** 10.3389/fpls.2024.1399718

**Published:** 2024-07-09

**Authors:** Varun Arya, Srinivasa Narayana, Twinke Sinha, Aravindaram Kandan, Samantapudi Venkata Satyanarayana Raju

**Affiliations:** ^1^ Insects Molecular Biology Laboratory, Institute of Agricultural Sciences, Department of Entomology and Agricultural Zoology, Banaras Hindu University, Varanasi, Uttar Pradesh, India; ^2^ Indian Council of Agricultural Research-National Bureau of Agricultural Insect Resources, Bengaluru, Karnataka, India; ^3^ Insect Physiology and Toxicology Laboratory, Institute of Agricultural Sciences, Department of Entomology and Agricultural Zoology, Banaras Hindu University, Varanasi, Uttar Pradesh, India

**Keywords:** *Bactrocera dorsalis*, species-specific primers, qPCR, primer sensitivity, pest identification, phytosanitary measures, fruit fly

## Abstract

The oriental fruit fly, *Bactrocera dorsalis* (Hendel), is a significant economic and quarantine pest due to its polyphagous nature. The accurate identification of *B. dorsalis* is challenging at the egg, maggot, and pupal stages, due to lack of distinct morphological characters and its similarity to other fruit flies. Adult identification requires specialized taxonomist. Existing identification methods are laborious, time consuming, and expensive. Rapid and precise identification is crucial for timely management. By analyzing the variations in the mitochondrial cytochrome oxidase-1 gene sequence (Insect barcoding gene), we developed a species-specific primer (SSP), DorFP1/DorRP1, for accurate identification of *B. dorsalis*. The optimal annealing temperature for the SSP was determined to be 66°C, with no cross-amplification or primer-dimer formation observed. The SSP was validated with *B. dorsalis* specimens from various locations in northern and eastern India and tested for cross-specificity with six other economically significant fruit fly species in India. The primer specificity was further confirmed by the analysis of critical threshold (Ct) value from a qPCR assay. Sensitivity analysis showed the primer could detect template DNA concentrations as low as 1 pg/µl, though sensitivity decreased at lower concentrations. Sequencing of the SSP-amplified product revealed over >99% similarity with existing *B. dorsalis* sequences in the NCBI GenBank. The developed SSP reliably identifies *B. dorsalis* across all developmental stages and sexes. This assay is expected to significantly impact pest identification, phytosanitary measures, and eradication programs for *B. dorsalis*.

## 1 Introduction

In the contemporary era of globalization, the escalating trade of fruits and vegetables stands as a primary catalyst for the dissemination of invasive insect-pest species into newer biogeographic realms (Brockerhoff et al., 2010; Suckling et al., 2014). Currently, the phytosanitary infrastructure in many quarantine zones, scattered across numerous ports of entry within various countries, has gradually become outdated (Ielmini and Sankaran, 2021). Compounded by the effects of climate change, the propagation of invasive pest species has gained momentum, posing an augmented threat to ecosystems on a global scale (Skendžić et al., 2021). Notably, there has been a recent surge in the global status attainment of several invasive insect species, occurring at a notably accelerated pace compared to historical trends (Hurley et al., 2016; Roques et al., 2016). Among these invasive pests, the oriental fruit fly, *Bactrocera dorsalis* (Hendel) (Tephritidae: Diptera), holds particular economic significance and has firmly established within India (Khan et al., 2023). Originally documented in East India (Clarke et al., 2019), subsequent studies by Clarke et al. (2019) and Qin et al. (2018) have identified Southeast Asian countries, particularly India and Bangladesh, as its centers of origin. Initially absent from the United States of America (USA), Europe, New Zealand, and Australia, *B. dorsalis* was widely distributed across Asia (excluding Korea and Japan) and African nations during early 2022 (Mutamiswa et al., 2021). However, within a span of just two years, it has expanded its range to encompass 46 African, 41 Asian, and 4 Oceanian countries, as well as certain regions of the USA (EPPO, 2024) (**Supplementary Figure 1**, https://gd.eppo.int/taxon/DACUDO/distribution) (accessed on 9 March, 2024). *Bactrocera philippinensis*, *B. papaya*, and *B. invadens* were previously considered junior synonyms of *B. dorsalis* (Schutze et al., 2015; Zeng et al., 2019). Nonetheless, Drew and Hancock (2022) have delineated them as distinct entities from *B. dorsalis* based on differences in the shapes and dimensions of the glans and preglans appendix. The maggot of *B. dorsalis* poses the most significant threat, with adult females utilizing their spiny ovipositors to penetrate the fruit skin and laying eggs beneath the rind. Upon hatching, the maggots voraciously consume the soft fruit pulp in a gregarious manner, resulting in complete destruction. Subsequently, the maggots pupate in the soil, from which the adults emerge (Mutamiswa et al., 2021).

India encompasses diverse biogeographical and agro-ecological zones, harboring approximately 7-8% of the world’s species (Venkataraman, 2012), thus earning the status of ‘megadiverse’ country (Rewatkar, 2020). This rich biodiversity fosters favorable conditions for the establishment and adaptation of agricultural pest species, posing challenges for effective management. Sridhar et al. (2014) utilized the CLIMAX simulation model to investigate the impact of climate change on the potential range expansion of *B. dorsalis* within India. Their findings projected that by 2023, the northern states of India would become more conducive for the establishment of *B. dorsalis*. Additionally, Choudhary et al. (2019) forecasted that temperature shift would alter the host-pest dynamics of *B. dorsalis*, potentially leading to an increase in voltinism (1-2 higher number of generations than usual and 15-24% reduction in the generation time over the basal period), which may increase the infestation by approximately 5% in the mango crop in India by 2050. Presently, India faces a challenging scenario necessitating integrated pest management strategies with pest eradication efforts. The cornerstone of any successful management program lies in the accurate identification of the pest. Malavasi et al. (2000) documented the prolonged duration required for the identification of *B. carambolae* in Suriname, highlighting the detrimental consequences of delayed identification on eradication endeavors (van Sauers-Muller, 2008). Similar challenges have been encountered in other regions, such as the failure to eradicate *B. dorsalis* in French Polynesia (Leblanc et al., 2013) and *B. zonata* in Egypt (Suckling et al., 2016), attributing to misidentification and the limitations of the existing detection techniques. Deschepper et al. (2023) studied the migration pathways of *B. dorsalis* in the islands of the Indian Ocean: the western invasion pathway originating from the east African coast, covering Comoros, Mayotte, and Madagascar into the Mascarene islands (Reunion and Mauritius), showing low genetic diversity and a direct colonization from the Asian subcontinent, forming a distinct cluster. Such situations highlight the critical need for a rapid and accurate pest identification tool. One promising technology in this regard is the mitochondrial cytochrome oxidase-I (mtCOI)-based species-specific primer (SSP) for *B. dorsalis*, offering expedited and precise identification capabilities crucial for effective pest management and eradication initiatives.

In the Indian context, the infestation rate of mango fruits by *B. dorsalis* varied from 66.66% to 76.47% (Saji et al., 2023), with percent yield loss ranging from 38% to 45% (Hossain et al., 2020), and mango fruit loss percent ranging from 5% to 80% (Maruthadurai and Ramesh, 2019). Countries importing mangoes from India enforce stringent quarantine protocols, requiring fruit to undergo either irradiation or hot water treatment prior to export to eliminate all the stages of fruit flies. However, if pests are detected during interception, they are either subjected to expert identification or identified via molecular barcoding, both of which are labor-intensive and time-consuming processes. In India, *B. dorsalis*, exhibits overlapping host preferences and morphological similarities with closely related fruit fly species, including *B. zonata* and *B. correcta*. Despite minor difference in coloration pattern (Drew and Hancock, 1994), wing shape and venation (Schutze et al., 2012), and genital characteristics (Iwahashi, 2001), which may not be easily discernible to general ecologist, proper identification necessitates the expertise of a taxonomist. The discovery of a single larva in an export consignment necessitates the destruction of the entire shipment, incurring approximately US$39,000 per container, with exporters bearing the financial burden (Ndiaye et al., 2008). *B. dorsalis* incurs annual financial losses exceeding US$2 billion in Africa, attributable to widespread fruit damage, high management cost, phytosanitary regulations, and interceptions (Korir et al., 2015; CABI/EPPO, 2018). Therefore, precise identification of this pest at land or ports of entry, as well as during eradication efforts is imperative. The morphological characteristics of *B. dorsalis* overlaps with those of many closely related species within the *Bactrocera* clade (Tan et al., 2011), not all of which are economically significant.

Although DNA barcoding of the mtCOI sequence offers an alternative to insect identification (Frewin et al., 2013), it is time-consuming, expensive, and contingent upon sequence availability in various GenBank databases. In contrast, SSPs provide rapid positive, and sensitive identification, even with a trace DNA quantity. For instance, Jiang et al. (2013) developed a SSP for *B. correcta*, capable of identifying all life stages with a template DNA concentration as low as 1 ng/μl. Lantero et al. (2017) devised a SSP for *B. oleae* to explore predation by Carabid arthropod predators through trace *B. oleae* DNA detection in predator gut. Andrews et al. (2022) developed the SSPs for successfully identifying and differentiating four species within the *Ceratitis* complex (*C. capitata, C. cosyra, C. rosa*, and *C. quilicii*) to detection limits of 4 ng to 10 ng of DNA template from both larvae and adults, with applications in port inspections and fruit examination. The developed PCR-based SSP facilitates rapid species identification, irrespective of stage or sex, within two to three hours of post-DNA extraction. This diagnostic tool holds promise for pest detection in quarantine centers, newly invaded areas, and timely eradication programs. Inspired by the aforementioned research efforts, the present study aims at developing a SSP for *B. dorsalis* based on the mtCOI gene sequence from the Indian fruit fly populations. Validation will include diverse population samples from various locations within the country, cross-amplification tests with related fruit fly template DNA using standard PCR and qPCR assay, sensitivity assessment for low-quality DNA, and on-field specificity test.

## 2 Materials and methods

### 2.1 Collection of fruit flies

**Figure 1:**
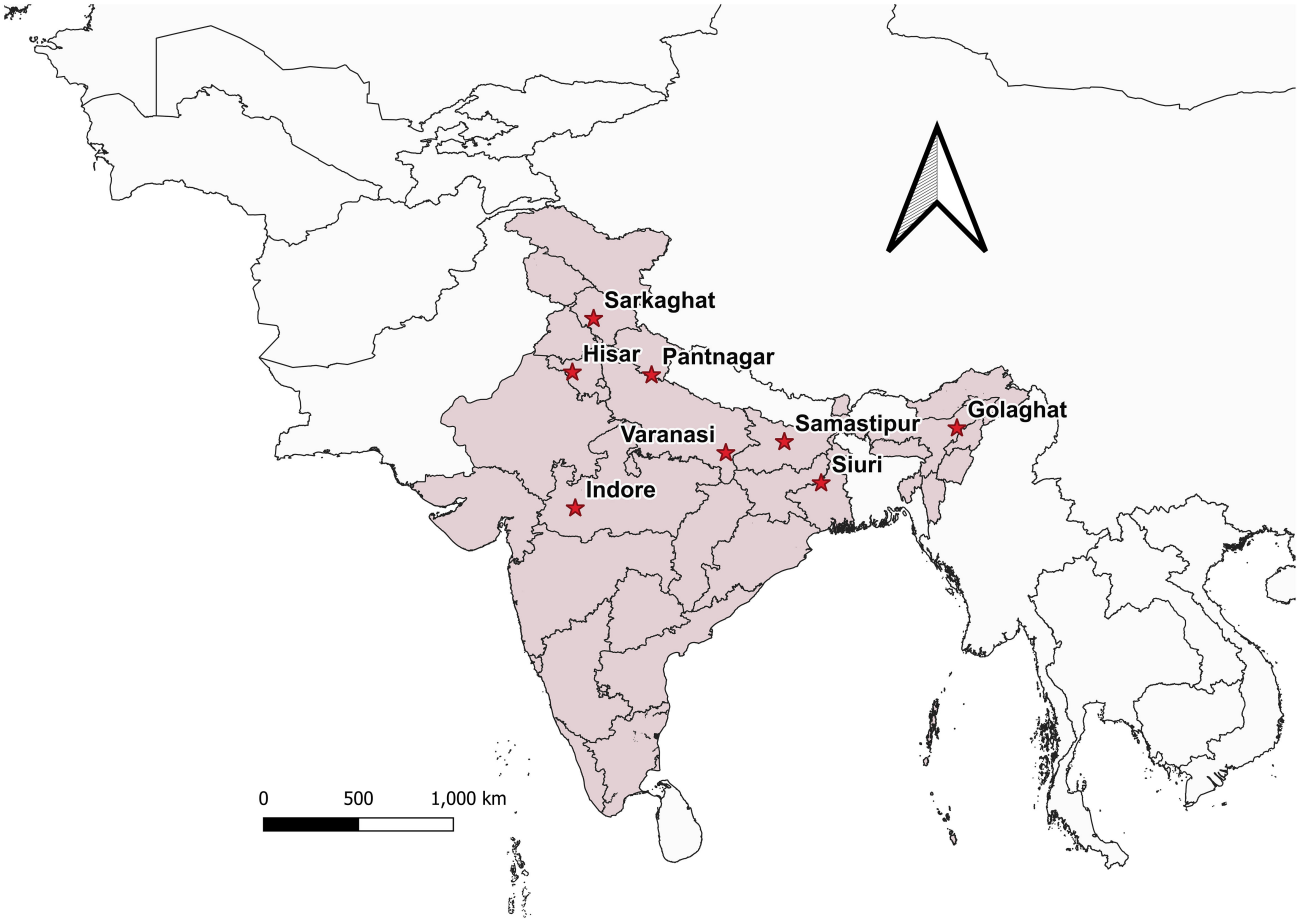
*Bactrocera dorsalis* collected from different locations in India is depicted in the map generated using QGIS 3.32.3. software.

*Bactrocera dorsalis* adults *♂* were collected from eight distinct Indian states during the year 2022 (**Figure 1**; **Table 1**), employing low-cost parapheromone bottle traps, prepared according to the specifications outlined by Arya et al. (2022). Methyl eugenol sourced from Sisco Research Laboratories Pvt. Ltd. (SRL) Mumbai, India, served as the attractant while Fipronil 5% SC (Adama Agadi^®^ SC) functioned as an insecticide. These traps were strategically positioned across diverse crop covers at a height of 1 m from the ground for a duration of two weeks. For each mango fruit displaying symptoms indicative of infestations such as internal rotting, pulpiness, dark lesions, splitting, and fluid exudation, five maggots were meticulously collected from the Horticultural Garden, Banaras Hindu University, Varanasi, Uttar Pradesh, India. Each fruit, enclosed within a sterilized glass beaker with a capacity of 1 liter and filled with sterilized soil to a depth of 5-7 cm, was covered with muslin cloth and maintained at a temperature of 27 ± 3°C. From the soil surrounding each fruit, five pupae were extracted, subsequently rinsed in distilled water, surface-sterilized using 90% ethanol, and preserved (**Supplementary Figure 2**). Upon emergence, the adults were harvested and stored for further examination. All specimens (maggots, pupae, and adults) were meticulously preserved in 90% ethanol and stored at -20°C until DNA extraction. The adults were scrutinized under a stereo-zoom microscope (Zoomstar-III, Dewinter), with their identities corroborated against existing taxonomic literature (David and Ramani, 2011; Leblanc et al., 2021) prior to molecular analysis. The immature stages of the verified *B. dorsalis* adults were subsequently subjected to DNA extraction.

**Table 1:** The collection sites along with the number of individuals of *Bactrocera dorsalis* collected.

S. No.	Collection sites	State code	Coordinates			No. of individuals collected (preserved for molecular analysis)
			Latitude (°)	Longitude (°)	Altitude (m)	
1.	Golaghat, Assam	AS	26.5239	93.9623	95	11(11)
2.	Hisar, Haryana	HR	29.1492	75.7217	215	8(8)
3.	Indore, Madhya Pradesh	MP	22.7196	75.8577	550	32(20)
4.	Pantnagar, Udham Singh Nagar, Uttarakhand	UK	29.0222	79.4908	243.8	8(8)
5.	Samastipur, Bihar	BR	25.8560	85.7868	47	38(20)
6.	Sarkaghat, Mandi, Himachal Pradesh	HP	31.6990	76.7324	911	43(20)
7.	Siuri, Birbhum, West Bengal	WB	23.9129	87.5268	41	24(20)
8.	Varanasi, Uttar Pradesh	UP	25.3176	82.9739	81	48(20)

### 2.2 DNA extraction and amplification of the mtCOI gene sequence

Genomic DNA extraction was performed from the mid and hind legs of the adult fruit flies (three individuals from each location), whereas entire maggots and pupae were utilized for immature stages, using the Qiagen DNeasy^®^ Blood and Tissue Kit, following the manufacturer’s instruction. The DNA quality of the samples was estimated using a Nanodrop 2000/2000c spectrophotometer (ThermoFisher Scientific), selecting samples with an A_260_/A_280_ ratio >1.8 for subsequent analysis. Amplification of the mtCOI gene sequence was performed employing universal barcode primers (LCO-1490: 5′-GGT CAA CAA ATC ATA AAG ATA TTG G-3′ and HCO-2198: 5′-TAA ACT TCA GGG TGA CCA AAA AATCA-3′) (Folmer et al., 1994). A 25 µl polymerase chain reaction (PCR) mixture was prepared, consisting of 12.5 µl Emerald Amp^®^ GT PCR master mix (TaKaRa), 8.5 µl of nuclease-free water, 0.5 µl of each forward and reverse primer, and 2 µl of the template DNA. PCR amplification was conducted using the Bio-Rad T100™ thermal cycler, with the following conditions: initial denaturation at 94°C for 5 min, 35 cycles of denaturation at 94°C for 30 s, annealing at 47°C for 40 s, initial extension at 72°C for 40 s followed by final extension at 72°C for 8 min. The PCR products were subjected to electrophoresis on a 2% TAE agarose gel (Merck-Milipore) stained with ethidium bromide and visualized under the Bio-Rad XR+ gel documentation system.

### 2.3 Development of the SSP for *B. dorsalis*

The complete mitochondrion genome and the mtCOI nucleotide sequences, representing distinct fruit fly species sourced from various geographic regions globally, were accessed from the NCBI GenBank database (https://www.ncbi.nlm.nih.gov/) (accessed on 4 November 2022) (**Supplementary Table S1**). Separate alignment of the mtCOI sequences and the complete mitochondrion genome was performed using the ‘ClustalW alignment’ tool integrated within the MEGA X software (Stecher et al., 2020), wherein scrutiny focused on discerning notable intra-specific similarities and inter-specific variabilities. A Neighbor-Joining (NJ) phylogenic tree (Saitou and Nei, 1987) was constructed from the aligned complete genomes using 1000 replicate bootstrap method (Felsenstein, 1985) and Kimura 2-parameter substitution model (Kimura, 1980), for similarity assessment and evolution analysis of the fruit flies. Subsequently, SSPs tailored for *B. dorsalis* were formulated utilizing the Primer3Plus tool (Untergasser et al., 2012), guided by predefined criteria encompassing marker length falling within the range of 18-30 bps, absorbance value (ΔG) below 9 kcal/mol, GC content ranging from 40% to 60%, and termination with a G/C nucleotide at the 3’ end (Tyagi et al., 2023). The specificity of the selected SSPs was corroborated through the NCBI primer BLAST utility (https://www.ncbi.nlm.nih.gov/tools/primer-blast/) (accessed on 2 August 2023). The selected SSPs were analyzed for potential specificity by checking the complementary sites of the forward and reverse primers in the pre-aligned fruit fly mtCOI sequences using the ‘ClustalW alignment’ tool in the MEGA X software. Assessment for potential hairpin structures, self-dimerization, and hetero-dimerization tendencies among the primers was conducted utilizing the Oligoanalyzer tool (http://www.idtdna.com/Home/Home.aspx) (accessed on 15 August 2023). The designed SSPs were synthesized by the M/S Eurofins Genomics India Pvt Ltd., Bengaluru, India, for subsequent experimental validation.

### 2.4 Selecting and validating the effective SSP through cross-amplification and sensitivity test

The designed SSPs underwent validation using *B. dorsalis* DNA extracted from various geographical regions via gradient PCR. PCR reaction volumes were minimized to 20 µl, comprising 10 µl of PCR master mix (Emerald Amp^®^ GT PCR master mix, TaKaRa), 7 µl of nuclease-free water, 0.5 µl of each primer (forward and reverse), and 2 µl of the template DNA. Thermal cycling conditions followed were as mentioned in the ‘DNA extraction and amplification of the mtCOI gene sequence’ section (2.2), with annealing temperature ranging from 55°C to 70°C for 40 s. Cross-amplification assessment was performed utilizing DNA templates from distinct fruit fly species: *B. zonata, B. correcta, Zeugodacus cucurbitae*, *Z. tau* and *B. digressa*, stored at -20°C in the ‘Insects Molecular Biology Laboratory’, Institute of Agricultural Sciences, Banaras Hindu University, Varanasi, Uttar Pradesh, India, in triplicate manner. Visualization of bands occurred on a 2% TAE agarose gel, and the most efficient SSP-amplified PCR products were subjected to Sanger sequencing (M/S Eurofins Genomics India Pvt. Ltd.) in duplicate to verify similarity with existing *B. dorsalis* mtCOI sequences submitted in the BOLD/NCBI GenBank libraries. Template DNA stock solutions from various species were prepared at a concentration of 50 ng/µl by dilution with nuclease-free water for cross-amplification studies. Marker efficiency was assessed using DNA from individual *B. dorsalis* specimens collected at each location (**Figure 1**; **Table 1**) including immature stage (maggots), in triplicate. Sensitivity testing involved diluting *B. dorsalis* DNA to concentration ranging from 60 ng/µl to 1 pg/µl, followed by PCR assay with consistent primer concentrations.

### 2.5 Validation of the primers via qualitative PCR assay

To further validate the specificity of the developed primer sets (DorFP1/DorRP1) for the target DNA sequence, a qualitative PCR (qPCR) assay was performed. The reactions were prepared in 96-well PCR plates with a total volume of 10 µl, comprising the following components: 5 µl of SYBR™ Green Real-Time PCR Master Mix (ThermoFisher Scientific), 1 µl each of forward and reverse primes, and 3 µl of template DNA. The template DNA from the fruit fly species used in the gradient PCR assay was included, along with the template DNA of *B. dorsalis* collected from UK, HP, AS, WB and BR, and two no-template controls (NTC) containing nuclease-free water instead of DNA, in triplicate. The reactions were conducted in a QuantStudio 5 Real-Time PCR systems (Applied Biosystems, Thermo Fischer Scientific, USA), following the thermal cyclic stages outlined in **Table 2**. Subsequent data analysis was performed with QuantStudio™ Design & Analysis Software v1.4 (Applied Biosystems, Thermo Fischer Scientific, USA). The amplified products were visualized in a 2% TAE agarose gel for possible amplifications. The cycle threshold (Ct) values for each sample, measured in triplicate, were analyzed statistically using a two-way analysis of variance (ANOVA) to determine if significant differences existed between the treatments. This was followed by Duncan’s multiple range test (DMRT) to compare the means (Duncan, 1955). The following statistical analysis was performed in the IBM SPSS Statistics for Windows version 22.0 statistical software package (IBM Corp.).

**Table 2:** Thermal cyclic stages of the qPCR assay.

Thermal cyclic stage	Steps	Temperature (°C)	Duration
Hold stage	Step 1	50.0	2 min
	Step 2	94.0	5 min
PCR stage (35 cycles)	Step 1	94.0	30 sec
	Step 2	66.0	1 min
Melt curve stage	Step 1	72.0	50 sec
	Step 2	66.0	1 min
	Step 3(dissociation)	95.0	1 sec

### 2.6 On-field specificity study of the developed SSPs

During the *kharif* season of 2023, fruits of bitter gourd (*Momordica charantia*), sponge gourd (*Luffa aegyptiaca*), tomato (*Solanum lycopersicum*), guava (*Psidium guajava*), and mango (*Mangifera indica*), exhibiting symptoms of fruit fly infestation, as described in the ‘Collection of fruit flies’ section (2.1), including the presence of actively feeding live maggots in the fruit pulp, were collected from the Banaras Hindu University campus. Each fruit was stored in a separate sterilized glass beaker (1 liter capacity), filled with sterilized soil to a height of 5-7 cm. Two to four maggots were meticulously harvested from each fruit and stored in 90% alcohol for DNA extraction, while the remaining maggots were allowed to feed and develop into adults to confirm the identity of the emerged fruit fly. The adults, once emerged, were collected and identified based on morphological characters, corroborated with available taxonomic literature by Leblanc et al. (2021) and David and Ramani (2011). DNA extraction of the preserved maggots was followed by PCR using the DorFP1/DorRP1 primers under the specific thermal cyclic conditions, and the amplified products were visualization on a 2% TAE agarose gel, as described in the ‘DNA extraction and amplification of the mtCOI gene sequence’ section (2.2), to demonstrate the practical application of the study.

## 3 Results

### 3.1 Estimation of DNA quality

**Figure 2:**
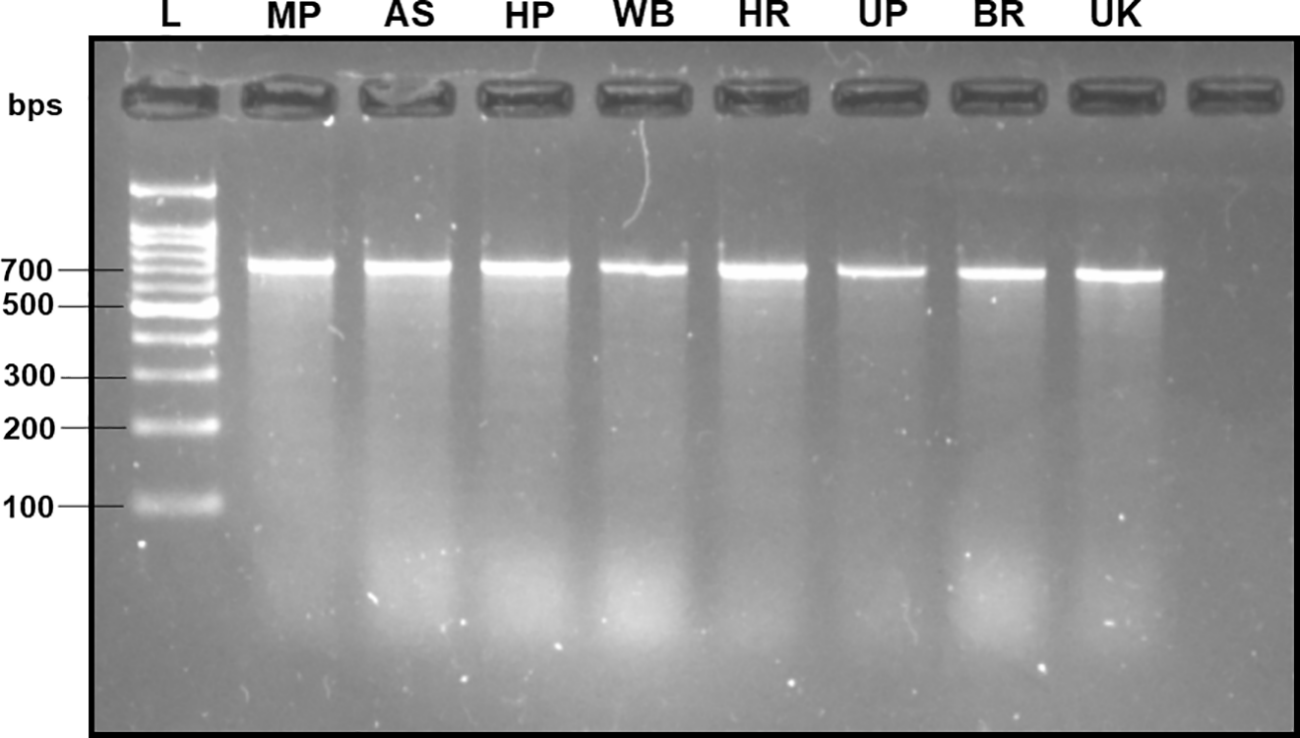
2% TAE agarose gel displaying the amplified mtCOI gene sequence of the *B. dorsalis* DNA via universal barcode primers (LCO-1490/HCO-2198) collected from different Indian states, showing a single clear band of ~700 bps size with reference to the molecular ladder, proving the use of high-quality extracted DNA. L: 100 bps molecular ladder (BR Biochem), *B. dorsalis* samples from MP, AS, HP, WB, HR, UP, BR, and UK, as per the state codes mentioned in **Table 1**.

The DNA extracted from the adult fruit flies exhibited high quality, characterized by a mean concentration of 164.70 ± 14.22 ng/µl, validated by the production of distinct band fragments at ~700 bps in a 2% TAE agarose gel (with reference to molecular ladder) through PCR amplification utilizing the universal barcode primer set (LCO-1490 and HCO-2198) (**Figure 2**). Therefore, within this investigation, the absence of PCR bands in assessing subsequent specificity and sensitivity cannot be attributed to an inadequate DNA template.

### 3.2 SSP selection based on cross-specificity test

**Figure 3:**
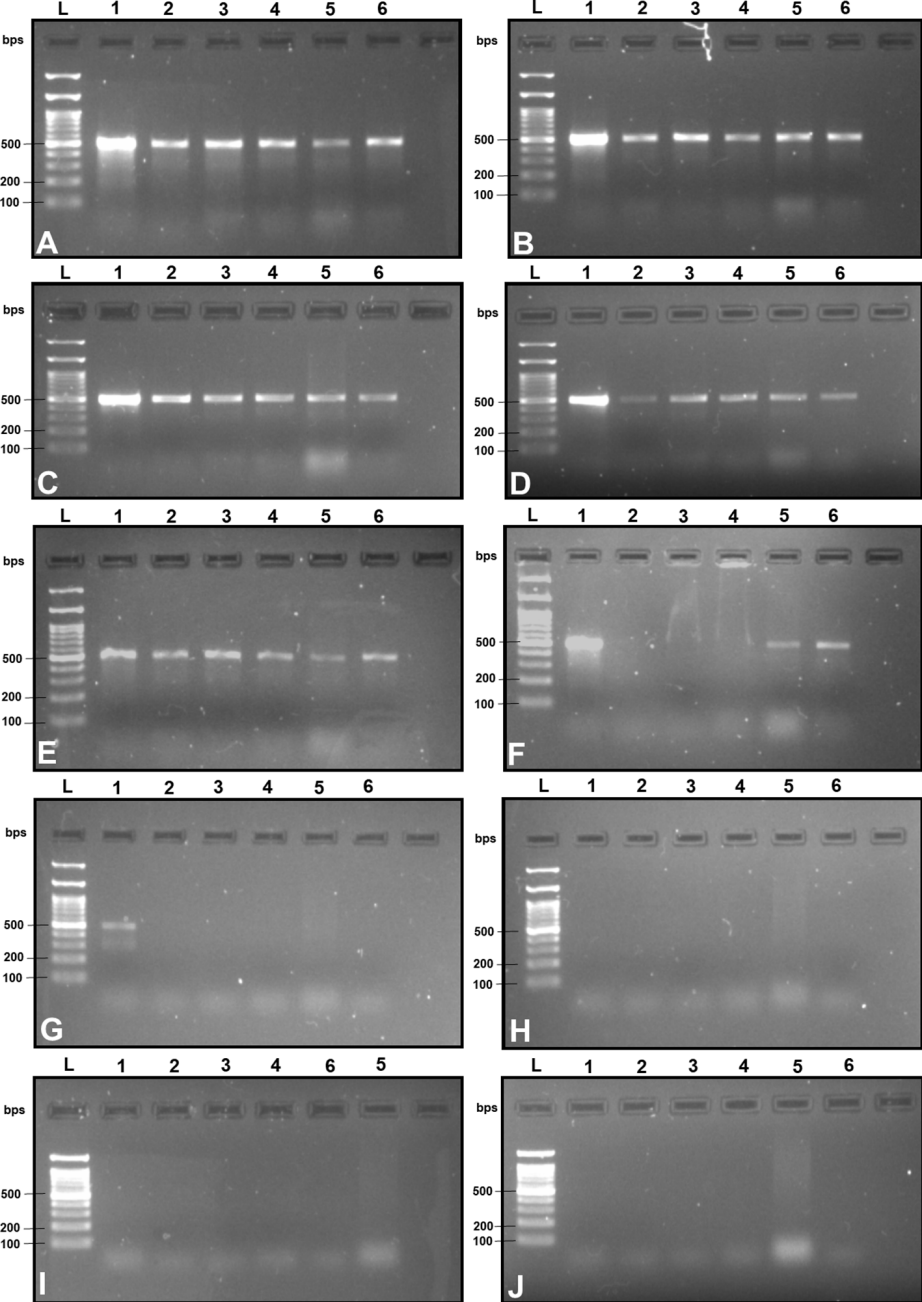
2% TAE agarose gel images displaying a replication of the cross-amplification test for the most effective SSP (DorFP1/DorRP1) at a range of annealing temperatures (°C) [**(A)**: 60, **(B)**: 61, **(C)**: 62, **(D)**: 63, **(E)**: 64, **(F)**: 65, **(G)**: 66, **(H)**: 67, **(I)**: 68 and **(J)**: 69] by representing a single clear band of 506 bps in each lane. L: 100 bps molecular ladder (BR Biochem), lane 1: *B. dorsalis*, lane 2: *B. zonata*, lane 3: *B. correcta*, lane 4: *Z. cucurbitae*, lane 5: *Z. tau* and lane 6: *B. digressa*. It represents the SSP showing no cross-amplification with the DNA templates of the tested fruit fly species at 66°C annealing temperature.

Upon examination of nucleotide polymorphism within various *B. dorsalis* DNA sequences sourced from NCBI GenBank accessions, three pairs of SSPs were formulated, denoted as DorFP1/DorRP1, DorFP2/DorRP2, and DorFP3/DorRP3, yielding amplified product of 506, 296, and 196 bps, respectively (**Table 3**). Notably, DorFP1/DorRP1 (**Supplementary Figure 3**) exhibited optimal performance, demonstrating no cross-amplification across diverse fruit fly species under the PCR annealing temperature of 66°C, generating a 506 bps product, as depicted in **Figure 3** (**Table 4**). Triplicate replication of experiments consistently corroborated these findings. Analysis of the species-specific amplified product confirmed >99% similarity to existing *B. dorsalis* nucleotide sequences within the BOLD/NCBI GenBank database. The nucleotide sequences have been deposited in the NCBI GenBank database under the accession numbers PP479586 and PP479587.

**Table 3:** Details of the species-specific primer developed for *Bactrocera dorsalis.*

S. No.	Primer Name	Primer Sequence (5’➔3’)	Length (bp)	T_m_ (°C)	GC (%)	Amplified product size (bp)
1.	DorFP1	CACCAGCCATATTGTGAGCC	20	56.2	55	506
	DorRP1	GTGTTCAGCTGGAGGGGTAT	20	56.7	55	
2.	DorFP2	CACCCAGGAGCTTTAATCGGT	21	57.1	52.4	296
	DorRP2	GCTCCTCCGTGTGCAATAAC	20	56.3	55	
3.	DorFP3	GTGGATTTGGAAATTGACTTGT	22	54	40.9	196
	DorRP3	GCTCCTCCGTGTGCAATAAC	20	56.3	55	

Tm, Melting temperature; GC, Guanine-Cytosine; bp, base-pairs.

**Table 4:** Results of cross-amplification test of the SSP (DorFP1/DorRP1) observed at different annealing temperatures.

Primer used	Annealing temp (T_a_ °C)	Amplification/showing positive results					
		*Bactrocera dorsalis*	*Bactrocera zonata*	*Bactrocera correcta*	*Zeugodacus cucurbitae*	*Zeugodacus tau*	*Bactrocera digressa*
DorFP1/DorRP1	60	✓	✓	✓	✓	✓	✓
	61	✓	✓	✓	✓	✓	✓
	62	✓	✓	✓	✓	✓	✓
	63	✓	✓	✓	✓	✓	✓
	64	✓	✓	✓	✓	✓	✓
	65	✓	✗	✗	✗	✓	✓
	**66**	**✓**	**✗**	**✗**	**✗**	**✗**	**✗**
	67	✗	✗	✗	✗	✗	✗
	68	✗	✗	✗	✗	✗	✗
	69	✗	✗	✗	✗	✗	✗

**✓**: positive test, **✗**: negative test. The shaded region indicates 66 oC as the optimum annealing temperature for the primer DorFP1/DorRP1 at which no cross-amplification was observed.

### 3.3 Efficacy of SSP, phylogenic analysis and sensitivity test

**Figure 4:**
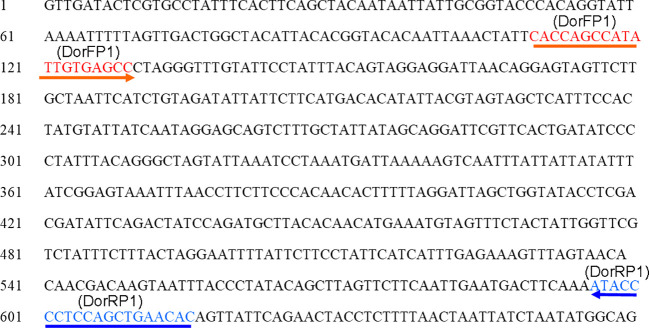
Alignment of the mtCOI sequence of *B. dorsalis* (NCBI Accession No. KM359573.1) with the locations of DorFP1/DorRP1 species-specific marker (direction: 5’➔3’, forward and reverse).

**Figure 5:**
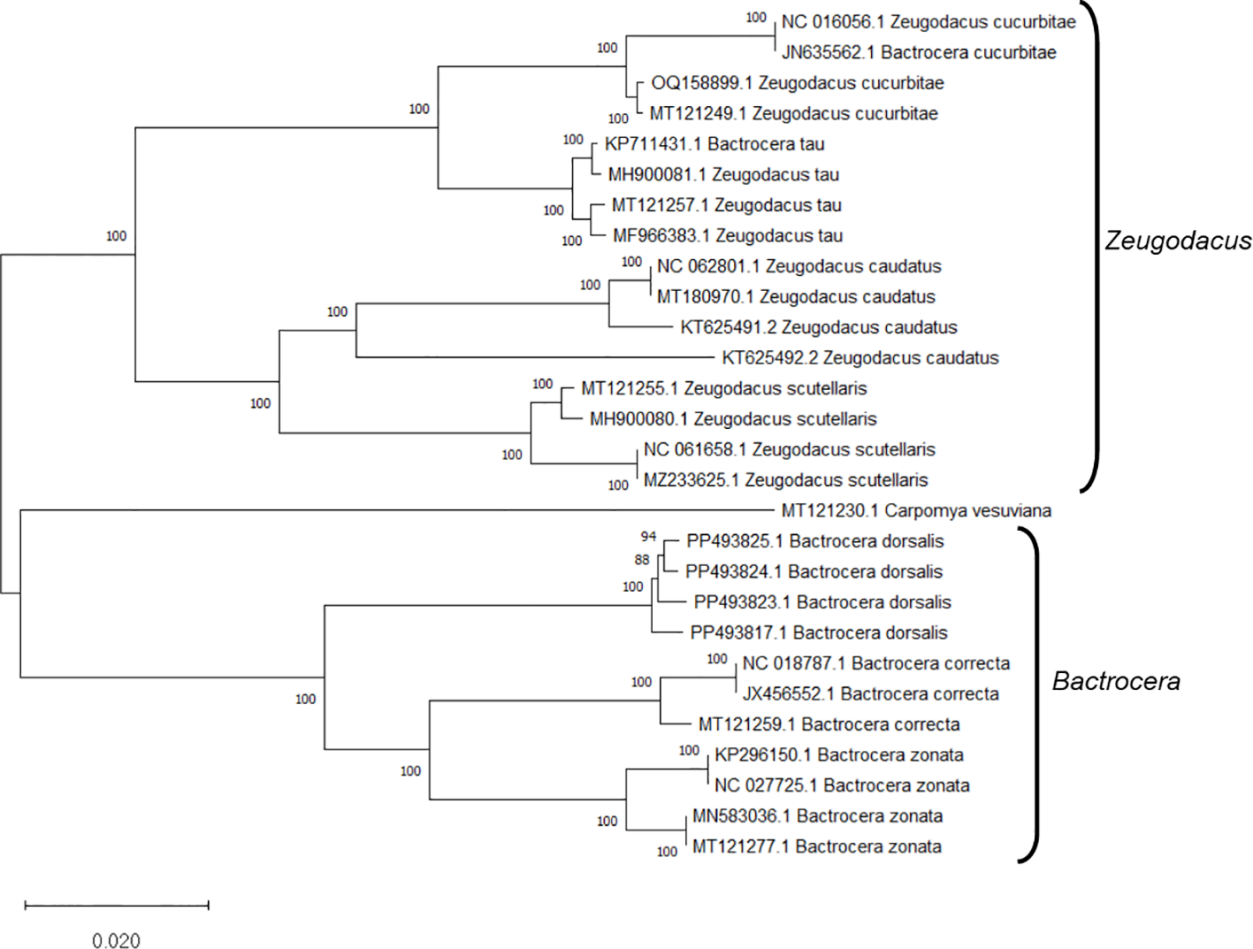
The Neighbor-Joining (NJ) phylogenetic tree constructed using the complete mitochondrial genome sequences of fruit flies, retrieved from NCBI GenBank, revealed robust clustering in bootstrap tests (1000 replicates). This analysis categorized the specimens into two distinct clades: *Bactrocera* and *Zeugodacus*.

**Figure 6:**
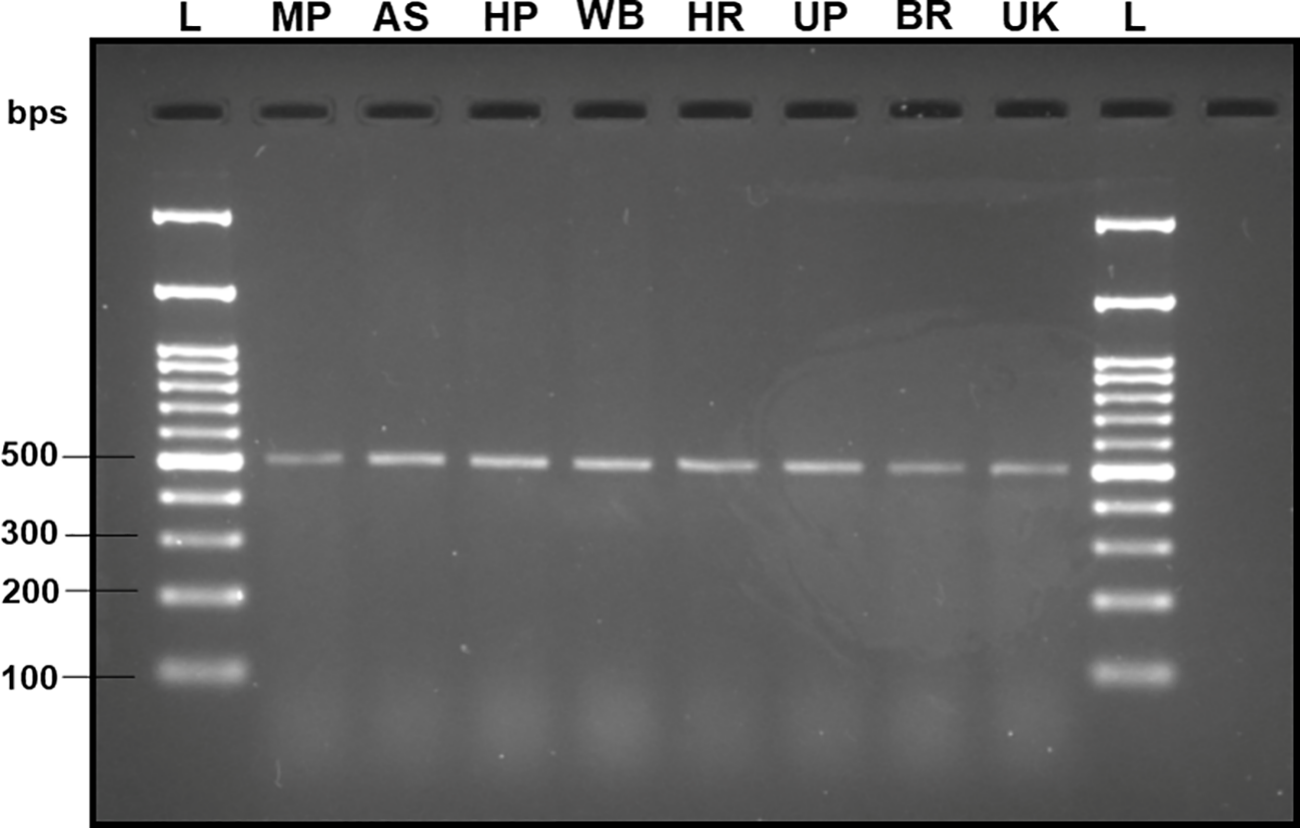
2% TAE agarose gel displaying validation of the specificity of the SSP (DorFP1/DorRP1) by successfully amplifying the *B. dorsalis* DNA samples collected from different geographical locations within India. Each lane represents a single-uniform band of 506 bps. L: 100 bps molecular ladder (BR Biochem), *B. dorsalis* samples from MP, AS, HP, WB, HR, UP, BR and UK, as per the state codes mentioned in **Table 1**.

**Figure 7:**
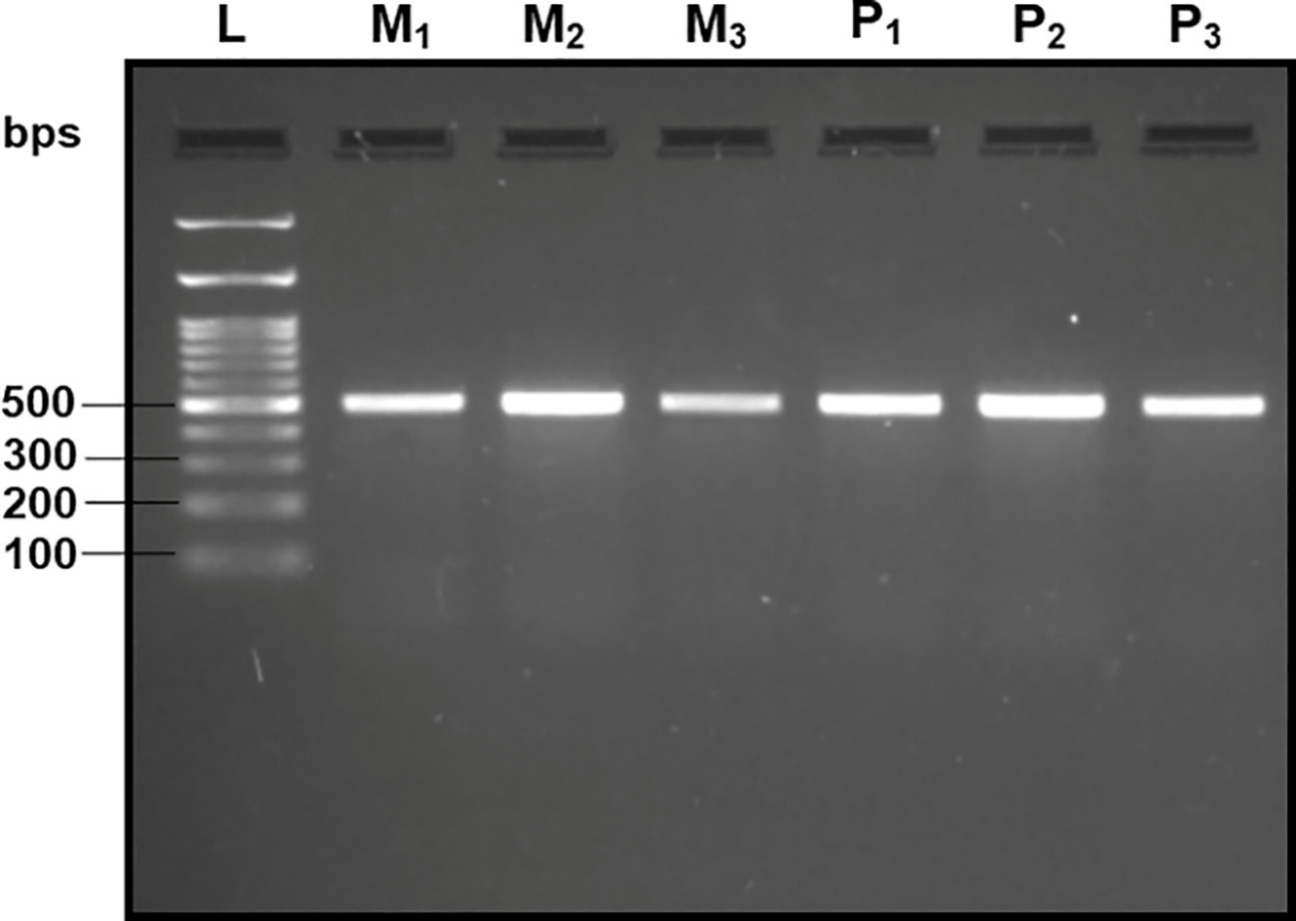
2% TAE agarose gel displaying the amplification by DorFP1/DorRP1 in the immature stages of the *B. dorsalis*. L: 100 bps molecular ladder (BR Biochem), M1, M2 and M3: three replicates of maggots, and P1, P2 and P3: three replicates of pupae.

**Figure 8:**
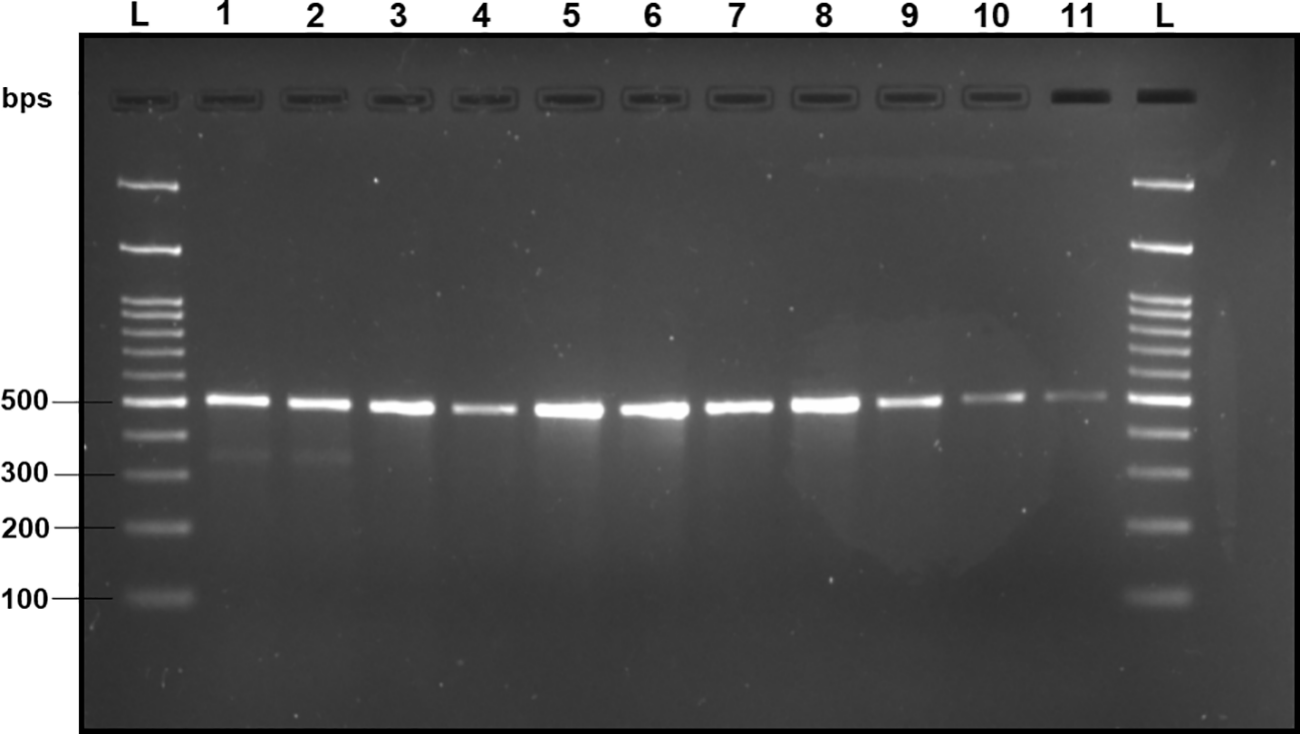
2% TAE agarose gel displaying the results of the sensitivity test of DorFP1/DorRP1 for different concentrations of DNA templates of *B. dorsalis.* L: 100 bps molecular ladder (BR Biochem), lane 1: 60 ng/µl, lane 2: 50 ng/µl, lane 3: 35 ng/µl, lane 4: 25 ng/µl, lane 5: 15 ng/µl, lane 6: 10 ng/µl, lane 7: 5 ng/µl, lane 8: 1 ng/µl, lane 9: 100 pg/µl, lane 10: 10 pg/µl and lane 11: 1 pg/µl.

Upon analyzing the primer specificity of the DorFP1/DorRP1 SSP using the NCBI primer BLAST utility, the primers were found to be highly specific to *B. dorsalis* nucleotide sequences submitted as the NCBI GenBank accessions, with >97% BLAST hits (number of *B. dorsalis* sequences in the database that have significant similarity with the product). This was further verified by performing nucleotide sequences alignment using the ‘ClustalW’ algorithm, depicting clear possible annealing of DorFP1/DorRP1 SSP within the *B. dorsalis* nucleotide sequences, and subsequent variations among the sequences of other selected species (**Figure 4**). The Neighbor-Joining (NJ) tree of the complete mitochondrial sequences of the fruit flies (**Figure 5**) clearly depicts *B. dorsalis* forming a distinct and a well-supported clade. The *B. dorsalis* clade has a high bootstrap value of 100, indicating a very high confidence level in the branching and distinguishing this species from others, thereby reinforcing the reliability of the phylogenetic separation and classification (Barr et al., 2021). *B. dorsalis* is closely related to *B. correcta*, as indicated by their adjacent positioning in the tree and the high bootstrap value of 100 for this branch. This shows significant genetic similarity, yet they are distinct enough to form separate clades. Additionally, *B. zonata* appears as another closely related species with equally high bootstrap support (100), suggesting a close evolutionary relationship within this group. The species arranged under the *Zeugodacus* clade are more distinct from *B. dorsalis* compared to the aforementioned species. The SSP demonstrated consistent amplification across all tested *B. dorsalis* specimens sourced from diverse geographical regions, as outlined in **Table 1**. As evident in **Figure 6**, the SSP efficiently amplified the 506 bps sequence from the mtCOI gene of *B. dorsalis*, yielding a single, uniform band at the 506 bps position relative to the molecular ladder in all the lanes of the gel. This outcome validated the SSP’s specificity, unaffected by variations in *B. dorsalis* DNA sequences collected from different regions across India. Additionally, the SSP exhibited amplification in template DNA extracted from immature *B. dorsalis* stages (maggots and pupae), as illustrated in **Figure 7**, underscoring its utility in identifying the pest during early development phases. In the sensitivity assessment, *B. dorsalis* DNA with a concentration of 184 ng/µl was serially diluted. Results indicated a diminishing intensity patterns of DNA bands at consistent concentrations and volumes of the SSP. Robust bands remained visible down to 10 pg/µl of the template DNA, with a faint band observed at 1 pg/µl DNA concentration, showcasing the high sensitivity of DorFP1/DorRP1 to minute amounts of template DNA, as depicted in the **Figure 8**. This underscores the SSP’s capability to amplify a trace quantities of template DNA effectively.

### 3.4 SSPs validation based on qPCR assay and on-field specificity test

**Figure 9:**
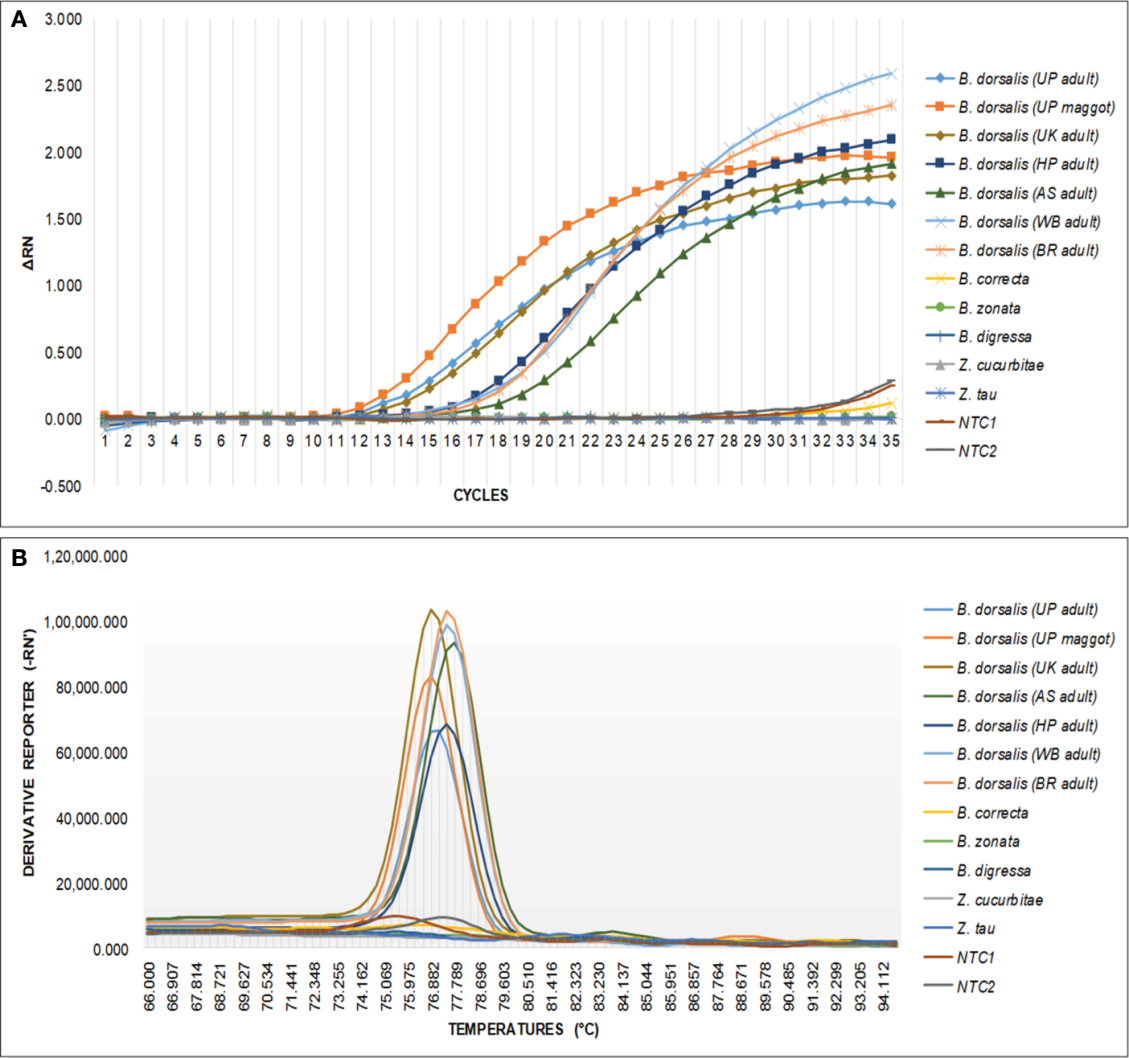
An optimized qPCR assay performed to assess the specificity of the DorFP1/DorRP1 SSPs using laboratory-extracted DNA templates from populations of *B. dorsalis* from UP (both maggot and adult), UK, HP, AS, WB, and BR. This was compared with *B. correcta, B. zonata, B. digressa, Z. cucurbitae*, and *Z. tau*, with inclusion of two NTC. **(A)** The graph illustrates the change in fluorescence signal (ΔRN) against the number of PCR cycles, representing successful amplification of samples containing *B. dorsalis* template DNA. **(B)** Melting curve analysis demonstrating species specificity of SSP for *B. dorsalis*, plotted against fluorescence derivative units (-RN’), and temperature (°C).

**Figure 10:**
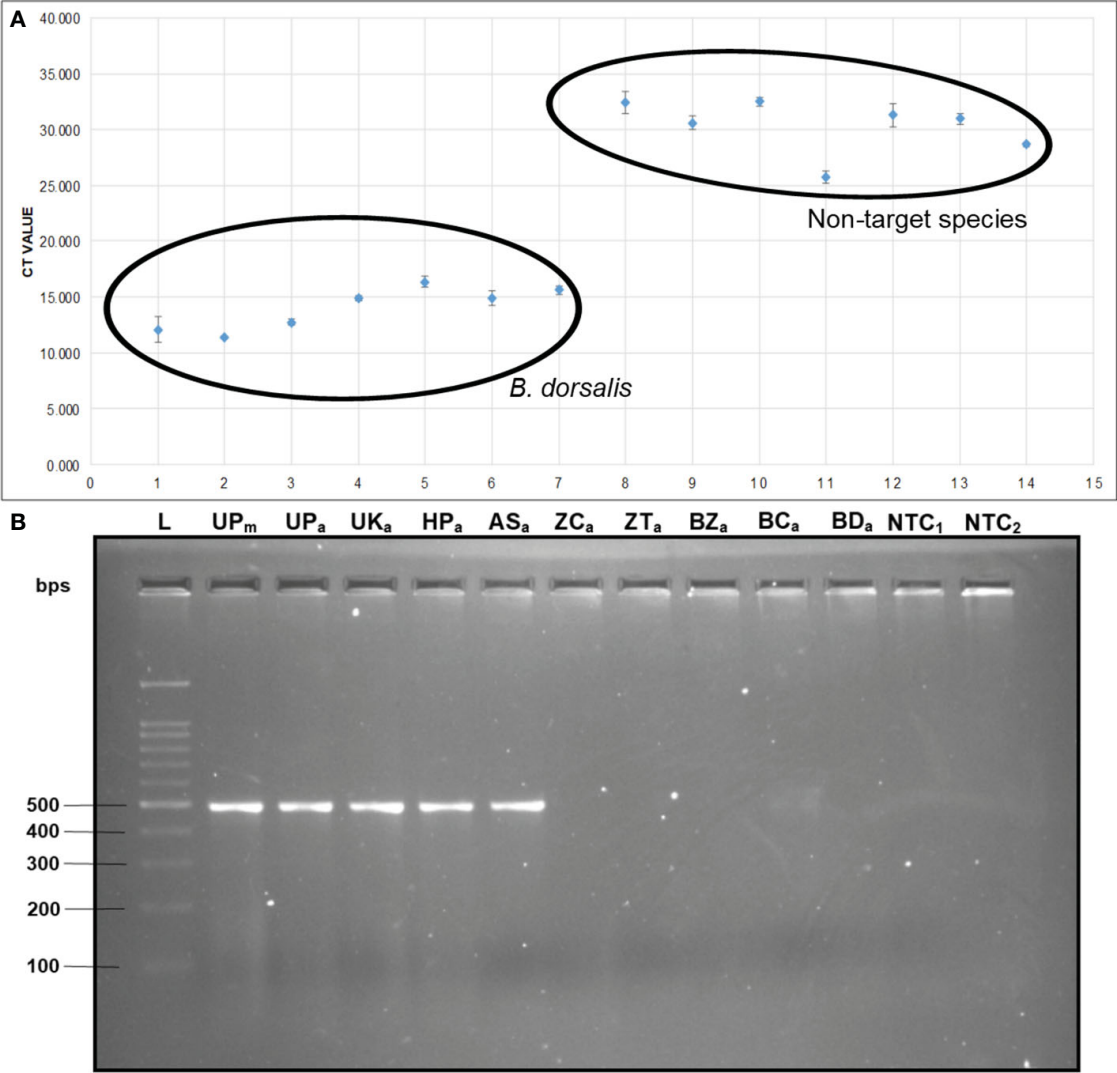
**(A)** Comparative analysis of the Ct values. The Ct values of *B. dorsalis* specimens were plotted against those of non-target fruit fly species. The specimens include: 1. *B. dorsalis* (UP maggot), 2. *B. dorsalis* (UP adult), 3. *B. dorsalis* (UK adult), 4. *B. dorsalis* (HP adult), 5. *B. dorsalis* (AS adult), 6. *B. dorsalis* (WB adult), 7. *B. dorsalis* (BR adult), 8. *B. correcta*, 9. *B. zonata*, 10. *B. digressa*, 11. *Z. cucurbitae*, 12. *Z. tau*, 13. NTC1, and 14. NTC2. **(B)** 2% TAE agarose gel of qPCR assay. The samples loaded in the following order: L: 100 bps molecular ladder (BR Biochem), lane 1: *B. dorsalis* (UP maggot), lane 2: *B. dorsalis* (UP adult), lane 3: *B. dorsalis* (UK adult), lane 4: *B. dorsalis* (HP adult), lane 5: *B. dorsalis* (AS adult), lane 6: *Z. cucurbitae*, lane 7: *Z. tau*, 8: lane 8: *B. zonata*, lane 9: *B. correcta*, lane 10: *B. digressa*, lane 11: NTC1, and lane 12: NTC2.

The qPCR analysis of various samples of *B. dorsalis*, including both maggot and adults from diverse geographical origins, exhibited robust amplification profiles (**Figure 9A**). The amplification commenced in the early exponential phase, typically between the 11^th^ and 15^th^ cycles, and progressed sharply before plateauing towards the end of the reaction. These findings suggest highly efficient and specific amplification of the target sequences. The non-target species (including *B. correcta, B. zonata, B. digressa, Z. cucurbitae*, and *Z. tau*) showed negligible amplification, highlighting the high specificity of the primer pair to *B. dorsalis*. Both the NTC exhibited no significant amplification throughout the cycles, confirming the absence of contamination and the specificity of the assay. In the qPCR melting curve analysis, SYBR green fluorescent dye was used to label double-stranded DNA. As the temperature increased, the DNA separated (melted), causing a decrease in florescence (Wittwer et al., 2024). The melting temperature was specific to the *B. dorsalis* DNA sequences. The peak began to develop at 72°C, marking SSP’s extension temperature, with distinct peaks observed around 75-77°C for *B. dorsalis* DNA, ensuring a successful amplification. No peaks observed for the DNA templates of other fruit fly species and the NTC (**Figure 9B**). The statistical analysis of the obtained Ct values showed a significant difference between the treatments (**Table 5**), with distinct groupings indicating which treatments are significantly different from each other. The Ct values represent the number of cycles required for the fluorescent signal to cross a threshold (Mishra et al., 2022). Samples containing the DNA template of *B. dorsalis* exhibited significantly low Ct values (11.383 to 16.347), indicating the presence of target DNA (**Table 6**). Conversely, the high Ct values of the non-target species and the NTCs confirmed the absence of contamination and non-specific amplification in the qPCR assay, demonstrating the high-sensitivity of the SSPs (**Figure 10A**). The specificity of the DorFP1/DorRP1 was further confirmed by visualizing the qPCR products on a 2% TAE agarose gel, which revealed clear, robust bands adjacent to the wells loaded with qPCR products containing *B. dorsalis* DNA template, in contrast to the qPCR products of non-template DNA (**Figure 10B**). In a standard PCR assay, the DorFP1/DorRP1 SSP accurately identified *B. dorsalis* among major economically important fruit flies infesting horticultural crops with 100% accuracy in a small study in the institution campus (**Table 7**). This experiment underscores the practical application and usefulness of the assay under real-field conditions.

**Table 5:** Two-way ANOVA table of Ct values for fruit fly DNA samples obtained in the qPCR assay.

Source of variation	Sum of squares (SS)	Degree of freedom (df)	Mean squares (MS)	F-values	*p-*values	F-critical values
Fruit fly template DNA	2939.8718	13	226.144	188.7612	2.7 × 10^-22^	2.1192
Replications	1.647	2	0.8235	0.6874	0.5118	3.369
Error (residual)	31.1491	26	1.198			
Total	2972.6679	41				

**Table 6:** The cycle threshold (Ct) values of reaction samples in the qPCR assay.

S. No.	Sample name	Mean Ct value ± S.E.
1.	*B. dorsalis* (UP maggot)	11.383 ± 0.06^a^
2.	*B. dorsalis* (UP adult)	12.09 ± 1.18^a^
3.	*B. dorsalis* (UK adult)	12.752 ± 0.25^a^
4.	*B. dorsalis* (WB adult)	14.887 ± 0.67^b^
5.	*B. dorsalis* (HP adult)	14.943 ± 0.22^b^
6.	*B. dorsalis* (BR adult)	15.638 ± 0.36^bc^
7.	*B. dorsalis* (AS adult)	16.347 ± 0.48^c^
8.	*Z. tau*	25.749 ± 0.58^d^
9.	NTC2	28.686 ± 0.18^e^
10.	*B. zonata*	30.631 ± 0.60^f^
11.	NTC1	30.995 ± 0.50^f^
12.	*B. digressa*	31.311 ± 1.01^f^
13.	*B. correcta*	32.474 ± 0.97^g^
14.	*Z. cucurbitae*	32.529 ± 0.37^g^

Following the Duncan's Multiple Range Test (DMRT), means were grouped based on their statistical differences. Superscripts were assigned according to pairwise comparisons, with means sharing the same superscript not being significantly different from each other at a significance level of 0.05.

**Table 7:** The results of on-field specificity test of DorFP1/DorRP1 SSP on fruit flies infesting major crops within the Banaras Hindu University campus, Varanasi, Uttar Pradesh, India.

Crops	Results of SSP analysis				Morphological identification of the adults
	+/-	+/-	+/-	+/-	
Bitter gourd (*Momordica charantia*)	–	–	–	–	*Z. cucurbitae*
	–	–	–	–	*Z. tau*
Sponge gourd (*Luffa aegyptiaca*)	–	–	–	–	*Z. cucurbitae*
Tomato (*Solanum lycopersicum*)	–	–	–	–	*Z. tau*
Guava (*Psidium guajava*)	–	–	–	–	*B. zonata*
	–	–	–	–	*B. correcta*
	+	+	+	+	*B. dorsalis*
Mango (*Mangifera indica*)	+	+	+	+	*B. dorsalis*
	–	–	–	–	*B. zonata*

## 4 Discussion

*Bactrocera dorsalis* stands as a paramount economic concern within the agricultural landscape of India, exhibiting a voracious appetite for a wide array of fruits and vegetable crops (Jena et al., 2022). Accurate identification of this species represents a pivotal challenge for the effective implementation of diverse management strategies. The phylogenic analysis using the NJ tree is particularly useful for constructing phylogenetic trees when sequence divergence is relatively low (Pearson et al., 1999). Analyzing the NJ phylogenic tree helped to identify clades that distinctly separate *B. dorsalis* from other species, focusing of branches/nodes having high bootstrap support. This facilitated the examining of aligned sequences corresponding to the branches in the NJ tree to locate unique genetic regions. The SSP developed in this investigation demonstrated precise identification of *B. dorsalis* specimens obtained from different Indian states, including the immature stages, even with template DNA concentration as low as 1 pg/µl, readily discernible on a 2% TAE agarose gel without necessitating further downstream analysis or sequencing. Dilution of the template DNA to 100 fg/µl and 10 fg/µl failed to yield any amplification, thereby establishing the lower limit of detection to 1 pg/µl. Notably, this method expedites species confirmation within a short timeframe post-DNA extraction, thereby mitigating temporal, financial, and resource constraints associated with conventional molecular identification via DNA barcoding. The selected primer pair demonstrated proficiency in generating a relatively larger amplicon size (506 bps) compared to alternative PCR-based SSPs developed for fruit flies (Jiang et al., 2013, Jiang et al., 2014; Afroza et al., 2022). Analysis of the primer-amplified product derived from diverse *B. dorsalis* mtCOI nucleotide sequences (**Supplementary Table 1**) utilizing the ‘Sequence Manipulation Suite’ (Stothard, 2000) unveiled 21 restriction sites for nine restriction endonucleases within the amplified region, attesting to its precision in delineating genetic variability and diversity within the species (Engels, 1981; Schulman, 2007). Moreover, the primer adeptly identified species-specific DNA fragments within the mtCOI sequence of *B. dorsalis* specimens collected across a wide elevational range within India, from low altitudes (≈47 m) to some of the highest elevations conducive to fruit fly survival (≈911 m) (**Table 1**), unaffected by genetic structural alterations, despite reports of substantial genetic variation within *B. dorsalis* mtCOI gene sequences across elevations (Shi et al., 2005; Liu et al., 2007). This underscores the potential conservation of the amplicon region within the species, furnishing a valuable tool for deciphering gene flow dynamics and enhancing comprehension of the genetic architecture of pest populations within the country (Manel et al., 2003; Adams et al., 2014). Furthermore, this knowledge can inform strategies for delineating reinvasion pathways and facilitate population categorization for effective management as discrete eradication units (Savidge et al., 2012).

Numerous contemporary methodologies have emerged for fruit fly identification, encompassing diverse assays such as real-time PCR/qualitative PCR (Koohkanzade et al., 2018), multiplex PCR (Chen et al., 2016), loop-mediated isothermal amplification (LAMP) (Blacket et al., 2020), restriction fragment length polymorphism (RFLP) (Barr et al., 2006), simple-sequence repeat (SSR) markers (Ding et al., 2018), and conventional PCR (Jiang et al., 2013), each offering unique applications and advantages. Notably, Rizzo et al. (2024) recently devised a qPCR assay targeting species-specific primers derived from mtCOI gene sequences to discriminate *B. dorsalis*, catering specifically to the prevailing pest dynamics in Europe. However, in the Indian context, qPCR facilities are not universally accessible, particularly in remote quarantine centers; nonetheless, conventional PCR remains ubiquitous in molecular research laboratories nationwide. The assay herein was meticulously developed to facilitate easy adoption and widespread dissemination of technology throughout the country. Furthermore, the efficacy of the developed SSP was rigorously evaluated across diverse haplotypic populations of *B. dorsalis* from India, improving the robustness of the primers. Despite this validation, cross-amplification testing of the *B. dorsalis* SSP was limited to some major economically significant fruit fly species exhibiting comparable infestation levels, shared host plants, and ecological niches within the region. The results were supported by Ct value analysis performed using the qPCR assay, showing significantly lower and consistent Ct values for *B. dorsalis* compared to other non-specific fruit flies. The Ct value inversely correlates with target DNA concentration (Rabaan et al., 2021) and leads to reproducible values across replicates (Svec et al., 2015), which is important for reliable quantification. In the present experiment, the lowest Ct value (11.383 ± 0.06) was obtained for the highest concentration of template DNA (*B. dorsalis* maggot: 210 ng/µl). The Ct values for *B. dorsalis* ranged closely from 11.38 to 16.34, compared to non-specific fruit flies, thereby supporting to the specificity of the SSP. According to melting curve analysis, the SSP produced a single peak corresponding to the specific target sequence, whereas multiple peaks can indicate non-specific amplification (Rodríguez et al., 2015). The *Bactrocera dorsalis* complex comprises approximately 79 species, delineated through comprehensive analysis incorporating morphological traits of adults and larvae, host-plant association, tissue-enzyme electrophoresis, morphometrics of adult male and female genitalia, male pheromone chemistry and molecular analysis (Drew and Hancock, 2022). Within the *dorsalis* complex, several species of the fruit fly have been documented in India, including *B. amarambalensis* Drew, *B. andamanensis* Kapoor, *B. carambolae* Drew & Hancock*, B. caryeae* Kapoor*, B. dorsalis* Hendel, *B. melastomatos* Drew & Hancock, *B. merapiensis* Drew & Hancock*, B. neoarecae* Drew*, B. paraverbascifoliae* Drew*, B. ranganathi* Drew & Romig*, B. syzygii* White & Tsuruta*, B. thailandica* Drew & Hancock, *B. verbascifoliae* Drew, and *B. vishnu* Drew and Hancock. However, of these, only *B. carambolae, B. caryeae* and *B. dorsalis* have been identified as significant agricultural pests (Vasudha et al., 2019; Drew and Hancock, 2022). *B. caryae* have been reported in the southern regions of India, spanning Goa, Karnataka, Tamil Nadu, and Kerala (Ramani et al., 2008), while *B. carambolae* has been observed in Meghalaya (Manger et al., 2018) and the Andaman and Nicobar Islands (Vasudha et al., 2019). Due to their geographical spread, economic importance, and absence in the sampled location, the aforementioned fruit fly species were excluded from the present study. Upon analyzing the nucleotide sequence of the SSP-based amplified products generated from the study (NCBI accession numbers PP479587 and PP479586), it was observed that these sequences exhibited similarities with the existing nucleotide sequences of *B. carambolae* and *B. raiensis* (approximately 0.89% to 1.90% on the basis of number of hits) in the NCBI-BLASTn (https://blast.ncbi.nlm.nih.gov/Blast.cgi) (accessed on 20 February, 2024). Nonetheless, the SSP DorFP1/DorRP1 successfully distinguished *B. dorsalis* from other agriculturally important fruit fly species found in India. Further analysis is warranted to differentiate the pest within the *dorsalis* complex. During the on-field experiment to analyze the specificity of the developed SSP for *B. dorsalis*, it was observed that individuals of a single fruit fly species emerged from a single fruit, whereas individuals of different species emerged from different fruits on the same tree (e.g., *B. dorsalis*, *B. zonata*, and *B. correcta* from guava). The likely reason for this is the deposition of oviposition deterrents on the fruit by the adult female of the fruit fly species that first reached it, preventing oviposition not only by individuals of different species but also by other females of the same species (Mojdehi et al., 2022). This underscores the importance of the SSP in identifying the presence of *B. dorsalis* on fruits and studying its oviposition behavior and chemical markers, which may have significant implications for pest management in the near future (Scolari et al., 2021).

India holds a prominent position globally as a competitive producer and exporter of various horticulturally significant crops, notably mango and guava (Raman et al., 2023), with Middle Eastern and Western European countries comprising the primary market recipients (Thakor, 2019). The country boasts 73 plant quarantine stations situated at international airports, seaports, and land borders, overseeing the regulation of plant material movement into and within its borders (Sushil et al., 2022). Detection of fruit fly infective propagules, including eggs, maggots, overwintering pupae, and active adults, poses a formidable challenge, given the potential for internal damage to remain concealed until symptoms appear, often rendering intervention too late for consignment salvage (Louzeiro et al., 2021). Recent findings by Akbar et al. (2020) unveiled new hosts for *B. dorsalis* in the Kashmir valley, underscoring the species’ capacity for host range expansion with prolonged persistence in natural habitats. Effective management or eradication of *B. dorsalis* necessitates rigorous measures during active crop growing periods, coupled with stringent field hygiene and post-harvest protocols during off-season intervals. These strategies, augmented by swift identification and elimination of infective propagules from non-domestic commodities within commercial fruit cultivation zones, offer promising avenues for large-scale pest management. Consequently, the *B. dorsalis* SSP developed and scrutinized in this study holds substantial promise, poised to assume a pivotal role in an array of fruit fly management programs, domestically and potentially on an international scale.

## 5 Conclusion

In this study, we have developed a PCR-based SSP, designated as DorFP1/DorRP1, tailored for the swift and precise identification of *B. dorsalis*. The technology harnessed herein is distinguished by its simplicity, reliability, cost-effectiveness, and robust sensitivity, enabling accurate discrimination of *B. dorsalis* across various metamorphic stages and sexes. Our investigation rigorously characterized the SSP’s specificity, revealing no instances of cross-amplification in the examined species, at PCR annealing temperatures of 66°C, significantly low qPCR Ct values for the target species, while also demonstrating remarkable sensitivity to a DNA template concentration as low as 1 pg/µl. To advance scientific understanding, additional investigation is required to access cross-amplification potential of the SSP assay across fruit fly species from a broad spectrum of geographic regions, particularly those including within the *B. dorsalis* complex. Furthermore, we explored the utility of the SSP-amplified product, with an amplicon size of 506 bps, as a potential surrogate tool for gauging genetic diversity within the species. As such, we posit that DorFP1/DorRP1 holds significant promise for applications in quarantine operations and diversity studies, thereby fostering opportunities for assessing its efficacy across a broader spectrum of fruit fly species sourced from diverse geographic locations. The SSP assay is anticipated to provide valuable insights into tracking the uncontrollable dissemination of the Indian population of *B. dorsalis*, informing decision-making process concerning international trade regulation, and serving a pivotal tool for monitoring and detection purposes.

## Data availability statement

The original contributions presented in the study are included in the article/**Supplementary Material**. Further inquiries can be directed to the corresponding author.

## Ethics statement

The manuscript presents research on animals that do not require ethical approval for their study.

## Author contributions

VA: Conceptualization, Data curation, Formal analysis, Investigation, Methodology, Software, Writing – original draft. SN: Conceptualization, Data curation, Formal analysis, Investigation, Methodology, Project administration, Resources, Software, Supervision, Validation, Visualization, Writing – original draft, Writing – review & editing. TS: Formal analysis, Investigation, Methodology, Validation, Writing – review & editing. AK: Methodology, Resources, Validation, Writing – review & editing. SR: Project administration, Resources, Supervision, Validation, Writing – review & editing.

## References

[B1] Adams A. L. Van Heezik Y. Dickinson K. J. M. Robertson B. C. (2014). Identifying eradication units in an invasive mammalian pest species. Biol. Invasions 16, 1481–1496. doi: 10.1007/s10530-013-0586-9

[B2] Afroza S. Nomana M. S. Zhanga Y. Alid M. Y. Mahmude M. R. Lia Z. (2022). Species identification of economic important fruit flies based on DNA bacrcoding (mt DNA COI) and larvae based on species specific primers from central and south parts of Bangladesh. Malays. J. Sustain. Agric. 6, 110–116. doi: 10.26480/mjsa.02.2022.110.116

[B3] Akbar S. A. Nabi S. U. Mansoor S. Khan K. A. (2020). Morpho-molecular identification and a new host report of *Bactrocera dorsalis* (Hendel) from the Kashmir valley (India). Int. J. Trop. Insect Sci. 40, 315–325. doi: 10.1007/s42690-019-00083-w

[B4] Andrews K. J. Bester R. Manrakhan A. Maree H. J. (2022). A multiplex PCR assay for the identification of fruit flies (Diptera: Tephritidae) of economic importance in South Africa. Sci. Rep. 12, 13089. doi: 10.1038/s41598-022-17382-x 35906478 PMC9338231

[B5] Arya V. Srinivasa N. Tyagi S. Raju S. V. S. (2022). A guide to prepare cue-lure for *Bactrocera cucurbitae* (Coquillett) management in cucurbits. Indian Entomologist 3, 45–47.

[B6] Barr N. B. Copeland R. S. De Meyer M. Masiga D. Kibogo H. G. Billah M. K.. (2006). Molecular diagnostics of economically important *Ceratitis* fruit fly species (Diptera: Tephritidae) in Africa using PCR and RFLP analyses. Bull. Entomol. Res. 96, 505–521. doi: 10.1079/BER2006452 17092362

[B7] Barr N. B. Hauser M. Belcher J. Salinas D. Schuenzel E. Kerr P.. (2021). Use of ITS-1 to identify *Bactrocera dorsalis* and *Bactrocera occipitalis* (Diptera: Tephritidae): a case study using flies trapped in California from 2008 to 2018. Fla. Entomol. 104, 96–106. doi: 10.1653/024.104.0205

[B8] Blacket M. J. Agarwal A. Zheng L. Cunningham J. P. Britton D. Schneider I.. (2020). A LAMP assay for the detection of *Bactrocera tryoni* Queensland fruit fly (Diptera: Tephritidae). Sci. Rep. 10. doi: 10.1038/s41598-020-65715-5 PMC7293347 32533005

[B9] Brockerhoff E. G. Liebhold A. M. Richardson B. Suckling D. M. (2010). Eradication of invasive forest insects: concepts, methods, costs and benefits. N. Z. J. For. Sci. 40, S117–S135.

[B10] CABI/EPPO (2018). “ Bactrocera dorsalis ,” in Distribution maps of plant pests (CAB International). Wallingford. UK

[B11] Chen Y. Dominiak B. C. O’Rourke B. A. (2016). A single multiplex PCR reaction for distinguishing strains of Queensland fruit fly *Bactrocera tryoni* (Diptera: Tephritidae). Austral Entomol. 55, 316–323. doi: 10.1111/aen.12190

[B12] Choudhary J. S. Mali S. S. Mukherjee D. Kumari A. Moanaro L. Rao M. S.. (2019). Spatio-temporal temperature variations in MarkSim multimodel data and their impact on voltinism of fruit fly, *Bactrocera* species on mango. Sci. Rep.9 1), 9708. doi: 10.1038/s41598-019-45801-z PMC6609607 31273224

[B13] Clarke A. R. Li Z. H. Qin Y. J. Zhao Z. H. Liu L. J. Schutze M. K. (2019). *Bactrocera dorsalis* (Hendel) (Diptera: Tephritidae) is not invasive through Asia: It’s been there all along. J. Appl. Entomol. 143, 797–801. doi: 10.1111/jen.12649

[B14] David K. J. Ramani S. (2011). An illustrated key to fruit flies (Diptera: Tephritidae) from Peninsular India and the Andaman and Nicobar Islands. Zootaxa 3021, 1–31. doi: 10.11646/zootaxa.3021.1.1

[B15] Deschepper P. Vanbergen S. Zhang Y. Li Z. Hassani I. M. Patel N. A.. (2023). *Bactrocera dorsalis* in the Indian Ocean: A tale of two invasions. Evol. Appl. 16, 48–61. doi: 10.1111/eva.13507 36699130 PMC9850006

[B16] Ding S. Wang S. He K. Li F. Jiang M. (2018). PCR-based identification of fruit-flies using specific SSR sequences. Chin. J. Appl. Entomol. 55, 759–765. doi: 10.7679/j.issn.2095-1353.2018.092

[B17] Drew R. A. Hancock D. (1994). The *Bactrocera dorsalis* complex of fruit flies (Diptera: Tephritidae: Dacinae) in Asia. L. Bull. Entomol. Res. Suppl. Ser. 2, 1–68. doi: 10.1017/S1367426900000278

[B18] Drew R. A. I. Hancock D. L. (2022). Biogeography, speciation and taxonomy within the genus *Bactrocera* Macquart with application to the *Bactrocera dorsalis* (Hendel) complex of fruit flies (Diptera: Tephritidae: Dacinae). Zootaxa 5190, 333–360. doi: 10.11646/zootaxa.5190.3.2 37045165

[B19] Duncan D. B. (1955). Multiple range and multiple F tests. Biometrics 11, 1–42. doi: 10.2307/3001478

[B20] Engels W. R. (1981). Estimating genetic divergence and genetic variability with restriction endonucleases. Proc. Nat. Acad. Sci. 78, 6329–6333. doi: 10.1073/pnas.78.10.632 6273864 PMC349032

[B21] EPPO (2024). Bactrocera dorsalis . In: EPPO datasheets on pests recommended for regulation. Available online at: https://gd.eppo.int (Accessed February 29, 2024).

[B22] Felsenstein J. (1985). Confidence limits on phylogenies: an approach using the bootstrap. Evol. 39, 783–791. doi: 10.1111/j.1558-5646.1985.tb00420.x 28561359

[B23] Folmer O. Black M. Hoeh W. Lutz R. Vrijenhoek R. (1994). DNA primers for amplification of mitochondrial cytochrome c oxidase subunit I from diverse metazoan invertebrates. Mol. Mar. Biol. Biotechnol. 3, 294–299. doi: 10.1093/molbev/msx281 7881515

[B24] Frewin A. Scott-Dupree C. Hanner R. (2013). DNA barcoding for plant protection: applications and summary of available data for arthropod pests. CABI Rev., 1–13. doi: 10.1079/PAVSNNR20138018

[B25] Hossain M. S. Sarkar B. C. Hossain M. M. Mian M. Y. Rajotte E. G. Muniappan R.. (2020). Comparison of biorational management approaches against mango fruit fly (*Bactrocera dorsalis* Hendel) in Bangladesh. Crop Prot. 135, 104807. doi: 10.1016/j.cropro.2019.05.001

[B26] Hurley B. P. Garnas J. Wingfield M. J. Branco M. Richardson D. M. Slippers B. (2016). Increasing numbers and intercontinental spread of invasive insects on eucalypts. Biol. Invasions 18, 921–933. doi: 10.1007/s10530-016-1081-x

[B27] Ielmini M. R. Sankaran K. V. (2021). “Invasive alien species: A prodigious global threat in the anthropocene,” in Invasive alien species: observations and issues from around the world, vol. 1 . Eds. Pullaiah T. Ielmini M. R. (Wiley Blackwell publications, UK), 1–79. doi: 10.1002/9781119607045.ch1

[B28] Iwahashi O. (2001). Aedeagal length of the Oriental fruit fly, *Bactrocera dorsalis* (Hendel) (Diptera: Tephritidae), and its sympatric species in Thailand and the evolution of a longer and shorter aedeagus in the parapatric species of *B. dorsalis* . Appl. Entomol. Zool. 36, 289–297. doi: 10.1303/aez.2001.289

[B29] Jena M. K. Patel S. R. Sahoo S. (2022). Biological and morphometric studies of fruit flies infesting fruit crops with special reference to *Bactrocera dorsalis*: A Review. J. Emerg. Technol. Innov. Res. 9, b22–b34.

[B30] Jiang F. Li Z. H. Deng Y. L. Wu J. J. Liu R. S. Buahom N. (2013). Rapid diagnosis of the economically important fruit fly, *Bactrocera correcta* (Diptera: Tephritidae) based on a species-specific barcoding cytochrome oxidase I marker. Bull. Entomol. Res. 103, 363–371. doi: 10.1017/S0007485312000806 23458744

[B31] Jiang F. Li Z. H. Wu J. J. Wang F. X. Xiong H. L. (2014). A rapid diagnostic tool for two species of *Tetradacus* (Diptera: Tephritidae: *Bactrocera*) based on species-specific PCR. J. Appl. Entomol. 138, 418–422. doi: 10.1111/jen.12041

[B32] Khan M. H. Salman M. Shah S. J. A. (2023). Field evaluation of various food attractants for the fruit fly *Bactrocera* species in pear orchard. Appl. Entomol. Phytopathol. 91, 1–10. doi: 10.22092/jaep.2023.362258.1479

[B33] Kimura M. (1980). A simple method for estimating evolutionary rates of base substitutions through comparative studies of nucleotide sequences. J. Mol. Evol. 16, 111–120. doi: 10.1007/BF01731581 7463489

[B34] Koohkanzade M. Zakiaghl M. Dhami M. K. Fekrat L. Namaghi H. S. (2018). Rapid identification of *Bactrocera zonata* (Dip.: Tephritidae) using TaqMan real-time PCR assay. PloS One 13, e0205136. doi: 10.1371/journal.pone.0205136 30286152 PMC6171934

[B35] Korir J. K. Affognon H. D. Ritho C. N. Kingori W. S. Irungu P. Mohamed S. A.. (2015). Grower adoption of an integrated pest management package for management of mango-infesting fruit flies (Diptera: Tephritidae) in Embu, Kenya. Int. J. Trop. Insect Sci. 35, 80–89. doi: 10.1017/S1742758415000077

[B36] Lantero E. Matallanas B. Ochando M. D. Pascual S. Callejas C. (2017). Specific and sensitive primers for the detection of predated olive fruit flies, *Bactrocera oleae* (Diptera: Tephritidae). Span. J. Agric. Res. 15, e1002–e1002. doi: 10.5424/sjar/2017152-9920

[B37] Leblanc L. Hossain M. A. Momen M. Seheli K. (2021). New country records, annotated checklist and key to the dacine fruit flies (Diptera: Tephritidae: Dacinae: Dacini) of Bangladesh. Insecta Mundi 0880, 1–56. doi: 10.15468/dl.yurtw8

[B38] Leblanc L. Vargas R. I. Putoa R. (2013). From eradication to containment: invasion of French Polynesia by *Bactrocera dorsalis* (Hendel) (Diptera: Tephritidae) and releases of two natural enemies: a 17-year case study. Proc. Hawaii. Entomol. Soc 45, 31–43.

[B39] Liu J. Shi W. Ye H. (2007). Population genetics analysis of the origin of the Oriental fruit fly, *Bactrocera dorsalis* Hendel (Diptera: Tephritidae), in northern Yunnan Province, China. Entomol. Sci. 10, 11–19. doi: 10.1111/j.1479-8298.2006.00194.x

[B40] Louzeiro L. R. F. de Souza-Filho M. F. Raga A. Gisloti L. J. (2021). Incidence of frugivorous flies (Tephritidae and Lonchaeidae), fruit losses and the dispersal of flies through the transportation of fresh fruit. J. Asia-Pac. Entomol. 24, 50–60. doi: 10.1016/j.aspen.2020.11.006

[B41] Malavasi A. Sauers-Muller A. V. Midgarden D. Kellman V. Didelot D. Caplong P.. (2000). “Regional program for the eradication of the carambola fruit fly in South America,” in Area-wide control of fruit flies and other insect pests’. Joint proceedings of the international conference on area-wide control of insect pests, 28 May-2 June, 1998 and the Fifth International Symposium on Fruit Flies of Economic Importance, Penang, Malaysia, Penerbit Universiti Sains Malaysia, 1-5 June, 1998. 395–399).

[B42] Manel S. Schwartz M. K. Luikart G. Taberlet P. (2003). Landscape genetics: combining landscape ecology and population genetics. Trends Ecol. Evol. 18, 189–197. doi: 10.1016/S0169-5347(03)00008-9

[B43] Manger A. Behere G. T. Firake D. M. Sharma B. Deshmukh N. A. Firake P. D.. (2018). Genetic characterization of *Bactrocera* fruit flies (Diptera: Tephritidae) from Northeastern India based on DNA barcodes. Mitochondrial DNA Part A 29, 792–799. doi: 10.1080/24701394.2017.1357713 28758818

[B44] Maruthadurai R. Ramesh R. (2019). Pheromone technology for the management of economically important insect pests. (Technical Bulletin No: 67) (Goa, India: ICAR-Central Coastal Agricultural Research Institute, Ela, Old Goa-403 402), 42.

[B45] Mishra B. Ranjan J. Purushotham P. Saha S. Payal P. Kar P.. (2022). High proportion of low cycle threshold value as an early indicator of COVID-19 surge. J. Med. Virol. 94, 240–245. doi: 10.1002/jmv.27307 34460115 PMC8661879

[B46] Mojdehi M. R. A. Keyhanian A. A. Rafiei B. (2022). Application of oviposition deterrent compounds for the control of olive fruit fly, *Bactrocera oleae* Rossi.(Dip. Tephritidae) control. Int. J. Trop. Insect Sci. 42, 63–70. doi: 10.1007/s42690-021-00518-3

[B47] Mutamiswa R. Nyamukondiwa C. Chikowore G. Chidawanyika F. (2021). Overview of oriental fruit fly, *Bactrocera dorsalis* (Hendel) (Diptera: Tephritidae) in Africa: From invasion, bio-ecology to sustainable management. Crop Prot. 141, 105492. doi: 10.1016/j.cropro.2020.105492

[B48] Ndiaye M. Dieng E. O. Delhove G. (2008). Population dynamics and on-farm fruit fly integrated pest management in mango orchards in the natural area of Niayes in Senegal. Pest Manage. Horti. Ecosyst. 14, 1–8.

[B49] Pearson W. R. Robins G. Zhang T. (1999). Generalized neighbor-joining: more reliable phylogenetic tree reconstruction. Mol. Biol. Evol. 16, 806–816. doi: 10.1093/oxfordjournals.molbev.a026165 10368958

[B50] Qin Y. J. Krosch M. N. Schutze M. K. Zhang Y. Wang X. X. Prabhakar C. S.. (2018). Population structure of a global agricultural invasive pest, *Bactrocera dorsalis* (Diptera: Tephritidae). Evol. Appl. 11, 1990–2003. doi: 10.1111/eva.12701 30459843 PMC6231469

[B51] Rabaan A. A. Tirupathi R. Sule A. A. Aldali J. Mutair A. A. Alhumaid S.. (2021). Viral dynamics and real-time RT-PCR Ct values correlation with disease severity in COVID-19. Diagnostics 11, 1091. doi: 10.3390/diagnostics11061091 34203738 PMC8232180

[B52] Raman M. S. Pant D. K. Singh A. Kumar R. (2023). Competitiveness of fruits’ and vegetables’ exports from India. Econ. Aff. 68, (3) 1379–1386. doi: 10.46852/0424-2513.3.2023.4

[B53] Ramani S. David K. J. Viraktamath C. A. Kumar A. R. V. (2008). Identity and distribution of *Bactrocera caryeae* (Kapoor) (Insecta: Diptera: Tephritidae)—a species under the *Bactrocera dorsalis* complex in India. Biosystematica 2, 49–57.

[B54] Rewatkar V. K. (2020). India as mega biodiversity nation: a fantastic “ethnobotanical museum. Int. J. Res. Biosci. Agric. Technol. 16, 1–6.

[B55] Rizzo D. Zubieta C. G. Sacchetti P. Marrucci A. Miele F. Ascolese R.. (2024). Diagnostic tool for the identification of *bactrocera dorsalis* (Hendel) (Diptera: tephritidae) using real-time PCR. Insects 15, 44. doi: 10.3390/insects15010044 38249050 PMC10815988

[B56] Rodríguez A. Rodríguez M. Córdoba J. J. Andrade M. J. (2015). “Design of primers and probes for quantitative real-time PCR methods,” in PCR primer design. Methods in molecular biology. Ed. Basu C. (Humana Press, New York, NY), 1275. doi: 10.1007/978-1-4939-2365-6_3 25697650

[B57] Roques A. Auger-Rozenberg M. A. Blackburn T. M. Garnas J. Pyšek P. Rabitsch W.. (2016). Temporal and interspecific variation in rates of spread for insect species invading Europe during the last 200 years. Biol. Invasions 18, 907–920. doi: 10.1007/s10530-016-1080-y

[B58] Saitou N. Nei M. (1987). The neighbor-joining method: a new method for reconstructing phylogenetic trees. Mol. Biol. Evol. 4, 406–425. doi: 10.1093/oxfordjournals.molbev.a040454 3447015

[B59] Saji S. J. Hole U. B. Bagde A. S. Shinde U. S. (2023). Efficacy of insecticides and biopesticides against mango fruit fly, *Bactrocera dorsalis* Hendel (Diptera: Tephritidae). J. Pharm. Innov. 12, 4035–4039.

[B60] Savidge J. A. Hopken M. W. Witmer G. W. Jojola S. M. Pierce J. J. Burke P. W.. (2012). Genetic evaluation of an attempted *Rattus rattus* eradication on Congo Cay, US Virgin Islands, identifies importance of eradication units. Biol. Invasions 14, 2343–2354. doi: 10.1007/s10530-012-0233-x

[B61] Schulman A. H. (2007). Molecular markers to assess genetic diversity. Euphytica 158, 313–321. doi: 10.1007/s10681-006-9282-5

[B62] Schutze M. K. Aketarawong N. Amornsak W. Armstrong K. F. Augustinos A. A. Barr N.. (2015). Synonymization of key pest species within the *Bactrocera dorsalis* species complex (Diptera: Tephritidae): taxonomic changes based on a review of 20 years of integrative morphological, molecular, cytogenetic, behavioral and chemo ecological data. Syst. Entomol. 40, 456–471. doi: 10.1111/syen.12113

[B63] Schutze M. K. Krosch M. N. Armstrong K. F. Chapman T. A. Englezou A. Chomič A.. (2012). Population structure of *Bactrocera dorsalis s.s., B. papayae* and *B. philippinensis* (Diptera: Tephritidae) in southeast Asia: evidence for a single species hypothesis using mitochondrial DNA and wing-shape data. BMC Evol. Biol. 12, 1–15. doi: 10.1186/1471-2148-12-130 22846393 PMC3458884

[B64] Scolari F. Valerio F. Benelli G. Papadopoulos N. T. Vaníčková L. (2021). Tephritid fruit fly semiochemicals: current knowledge and future perspectives. Insects 12, 408. doi: 10.3390/insects12050408 33946603 PMC8147262

[B65] Shi W. Kerdelhue C. Ye H. (2005). Population genetics of the oriental fruit fly, *Bactrocera dorsalis* (Diptera: Tephritidae), in Yunnan (China) based on mitochondrial DNA sequences. Environ. Entomol. 34, 977–983. doi: 10.1603/0046-225X-34.4.977

[B66] Skendžić S. Zovko M. Pajač Živković I. Lešić V. Lemić D. (2021). Effect of climate change on introduced and native agricultural invasive insect pests in Europe. Insects 12, 985. doi: 10.3390/insects12110985 34821786 PMC8619401

[B67] Sridhar A. Verghese A. Vinesh L. S. Jayashankar M. Jayanthi P. K. (2014). CLIMEX simulated predictions of Oriental fruit fly, Bactrocera dorsalis (Hendel) (Diptera: Tephritidae) geographical distribution under climate change situations in India. Curr. Sci. 12, 1702–1710. Available at: https://www.jstor.org/stable/24103005.

[B68] Stecher G. Tamura K. Kumar S. (2020). Molecular evolutionary genetics analysis (MEGA) for macOS. Mol. Biol. Evol. 37, 1237–1239. doi: 10.1093/molbev/msz312 31904846 PMC7086165

[B69] Stothard P. (2000). The sequence manipulation suite: JavaScript programs for analyzing and formatting protein and DNA sequences. Biotechniques 28, 1102–1104. doi: 10.2144/00286ir01 10868275

[B70] Suckling D. M. Kean J. M. Stringer L. D. Cáceres-Barrios C. Hendrichs J. Reyes-Flores J.. (2016). Eradication of tephritid fruit fly pest populations: outcomes and prospects. Pest Manage. Sci. 72, 456–465. doi: 10.1002/ps.2016.72.issue-3 25204807

[B71] Suckling D. M. Stringer L. D. Stephens A. E. Woods B. Williams D. G. Baker G.. (2014). From integrated pest management to integrated pest eradication: technologies and future needs. Pest Manage. Sci. 70, 179–189. doi: 10.1002/ps.3670 24155254

[B72] Sushil S. N. Joshi D. Roy S. Rao G. P. Pathak A. D. (2022). Plant quarantine regulations with reference to sugarcane in India: strengths and challenges. Sugar Tech 24, 1319–1329. doi: 10.1007/s12355-022-01125-3

[B73] Svec D. Tichopad A. Novosadova V. Pfaffl M. W. Kubista M. (2015). How good is a PCR efficiency estimate: Recommendations for precise and robust qPCR efficiency assessments. Biomol. Detect. Quantif. 3, 9–16. doi: 10.1016/j.bdq.2015.01.005 27077029 PMC4822216

[B74] Tan K. H. Tokushima I. Ono H. Nishida R. (2011). Comparison of phenylpropanoid volatiles in male rectal pheromone gland after methyl eugenol consumption, and molecular phylogenetic relationship of four global pest fruit fly species: Bactrocera invadens, B. dorsalis, B. correcta and *B. zonata* . Chemoecology 21, 25–33. doi: 10.1007/s00049-010-0063-1

[B75] Thakor N. J. (2019). Indian mango–production and export scenario. Peach 18, 0–12.

[B76] Tyagi S. Srinivasa N. Singh R. N. Vinay N. (2023). Species-specific markers for *Nilaparvata lugens* and *Sogatella furcifera* (Hemiptera: Delphacidae) based on mitochondrial cytochrome oxidase I. 3 Biotech. 13, 269. doi: 10.1007/s13205-023-03693-x PMC10335986 37449252

[B77] Untergasser A. Cutcutache I. Koressaar T. Ye J. Faircloth B. C. Remm M.. (2012). Primer3—new capabilities and interfaces. Nucleic Acids Res. 40, e115–e115. doi: 10.1093/nar/gks596 22730293 PMC3424584

[B78] van Sauers-Muller A. (2008). Carambola fruit fly situation in Latin America and the Caribbean. Proc. Carib. Food Crops Soc 44, (1) 135–144. doi: 10.22004/ag.econ.256497

[B79] Vasudha A. Ahmad A. Agarwal M. L. (2019). An overview of Indian dacine fruit flies (Diptera: tephritidae: dacinae: dacini). Int. J. Bio-Res. Stress Manage. 10, 491–506. doi: 10.23910/IJBSM/2019.10.5.2016

[B80] Venkataraman K. (2012). Biodiversity and its conservation. Proc. Natl. Acad. Sci. India B- Biol. Sci. 82, 271–282. doi: 10.1007/s40011-012-0096-z

[B81] Wittwer C. T. Hemmert A. C. Kent J. O. Rejali N. A. (2024). DNA melting analysis. Mol. Aspects Med. 97, 101268. doi: 10.1016/j.mam.2024.101268 38489863

[B82] Zeng Y. Reddy G. V. Li Z. Qin Y. Wang Y. Pan X.. (2019). Global distribution and invasion pattern of oriental fruit fly, *Bactrocera dorsalis* (Diptera: Tephritidae). J. Appl. Entomol. 143, 165–176. doi: 10.1111/jen.12582

