# Supplementary figures and images for: A simple PCR-based quick detection of the economically important oriental fruit fly, Bactrocera dorsalis (Hendel) from India
Source: Front Plant Sci. 2024 Jul 9;15:1399718. doi: 10.3389/fpls.2024.1399718 (PMC11263087; doi:10.3389/fpls.2024.1399718)

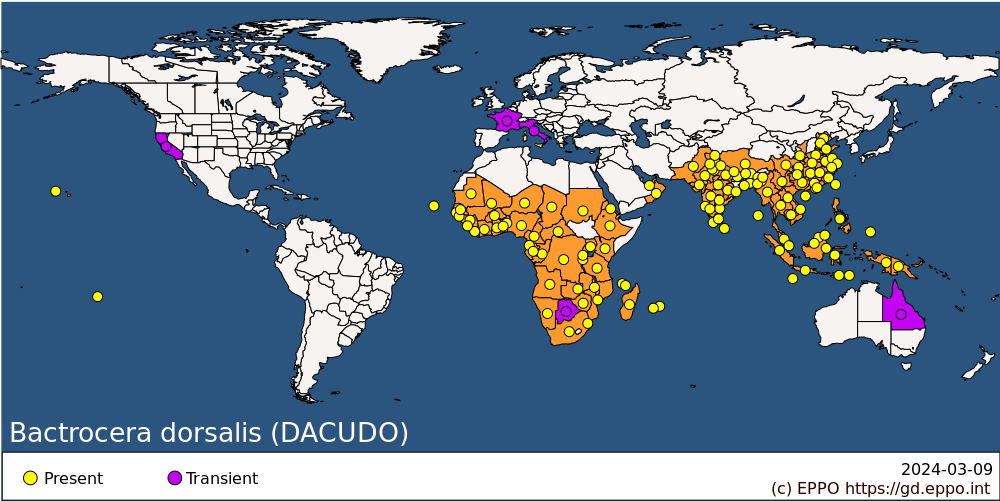

Supplement: Supplementary file 1 [file Image_1.png]

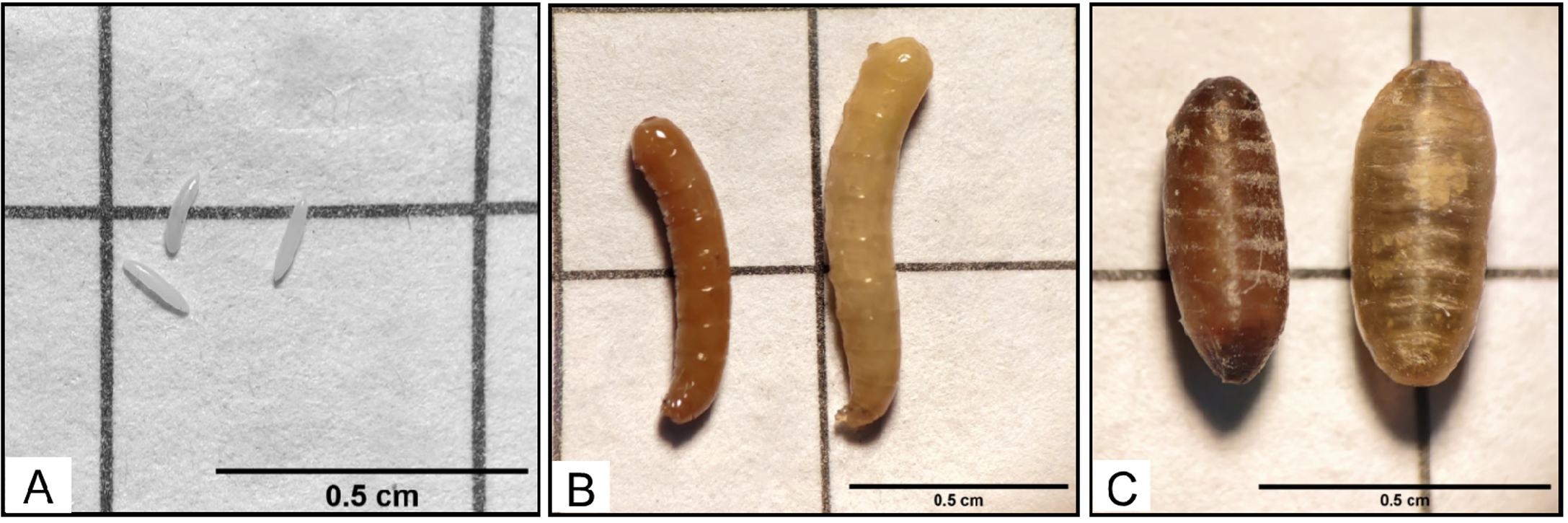

Supplement: Supplementary file 2 [file Image_2.tif]
