# Supplementary material for: A simple PCR-based quick detection of the economically important oriental fruit fly, Bactrocera dorsalis (Hendel) from India
Source: Front Plant Sci. 2024 Jul 9;15:1399718. doi: 10.3389/fpls.2024.1399718 (PMC11263087; doi:10.3389/fpls.2024.1399718)
Supplement: Supplementary file 3 [file Image_3.pdf]

```
                                      10        20        30        40        50        60
                              ....|....|....|....|....|....|....|....|....|....|....|....|
KM359573.1 Bactrocera dorsalis ------------------------------------------------------------
HQ446518.1 Bactrocera dorsalis ------------------------------------------------------------
OQ626326.1 Bactrocera dorsalis ------------------------GGATTCGGAATAATTTCCCATATTATTAGTCAAGAA
OP036560.1 Bactrocera dorsalis -----------------------AGGATTCGGAATAATTTCCCATATTATTAGTCAAGAA
OK175615.1 Bactrocera dorsalis ------------------------------------------------------------
MW032650.1 Bactrocera dorsalis ------------------------------------------------------------
MT257267.1 Bactrocera correcta --------CATTTTAATTTTACCAGGATTCGGAATAATTTCTCATATTATTAGTCAAGAA
MK053681.1 Bactrocera correcta ------------------------------------------------------------
KJ753908.1 Bactrocera correcta ------------------------------------------------------------
MN016970.1 Bactrocera correcta ------------------------------------------------------------
AY530905.1 Bactrocera correcta ------------------------------------------------------------
JQ692631.1 Bactrocera correcta ------------------------------------------------------------
MT257819.1 Bactrocera zonata --------CATTTTAATTCTACCAGGATTCGGAATAATTTCCCATATTATTAGTCAAGAA
MG881756.1 Bactrocera zonata ------------------------------------------------------------
JX965418.1 Bactrocera zonata ------------------------------------------------------------
MT257315.1 Bactrocera zonata --------CATTTTAATTCTACCAGGATTCGGAATAATTTCCCATATTATTAGTCAAGAA
MT258188.1 Bactrocera zonata --------CATTTTAATTCTACCAGGATTCGGAATAATTTCCCATATTATTAGTCAAGAG
KJ142766.1 Zeugodacus cucurbit --------------------------ATTCGGTATAATTTCTCATATTATTAGACAAGAA
MN016983.1 Zeugodacus cucurbit ------------------------------------------------------------
KP851001.1 Zeugodacus cucurbit ------------------------------------------------------------
OL701253.1 Zeugodacus cucurbit ------------------------------------------------------------
OK103985.1 Zeugodacus cucurbit ------------------------------------------------------------
MW300735.1 Zeugodacus cucurbit ------------------------------------------------------------
KT175576.1 Zeugodacus tau ------------------------------------------------------------
OL701270.1 Zeugodacus tau ------------------------------------------------------------
MT257465.1 Zeugodacus tau --------TATTTTAATTTTACCAGGATTTGGAATAATTTCTCACATTATTAGACAAGAA
MK249718.1 Zeugodacus tau ------------------------------------------------------------
KJ753948.1 Bactrocera tau ------------------------------------------------------------
MT456363.1 Bactrocera digressa --------TATTTTAATTCTACCAGGATTTGGAATAATTTCTCATATTATTAGTCAAGAA
MG683319.1 Bactrocera digressa ------------------------------------------------------------
GQ458048.1 Bactrocera caudata ------------------------------------------------------------
FJ903493.1 Bactrocera caudata ------------------------------------------------------------
JX559676.1 Bactrocera caudata ------------------------------------------------------------
KF659996.1 Bactrocera caudata ------------------------------------------------------------
MK125511.1 Zeugodacus caudatus ------------------------------------------------------------
AF423109.1 Bactrocera caudata ------------------------------------------------------------
MK053684.1 Bactrocera nigrofem ------------------------------------------------------------
MK660603.1 Bactrocera nigrofem ------------------------------------------------------------
MH172171.1 Bactrocera nigrofem ------------------------------------------------------------
KT594975.1 Bactrocera scutella GAAGTTTACATTTTAATTTTACCAGGATTCGGAATAATCTCCCATATTATTAGCCAAGAA
MK053674.1 Zeugodacus scutella ------------------------------------------------------------
KM024429.1 Bactrocera scutella ------------------------------------------------------------
KT588387.1 Zeugodacus scutella ------------------------------------------------------------
KF660074.1 Bactrocera scutella ------------------------------------------------------------
KJ753902.1 Bactrocera cilifera ------------------------------------------------------------
MH395849.1 Zeugodacus cilifer ------------------------------------------------------------
JX266417.1 Bactrocera cilifera ------------------------------------------------------------
KF660011.1 Bactrocera cilifera ------------------------------------------------------------
MF095190.1 Zeugodacus cilifer ------------------------------------------------------------

                                      70        80        90       100       110       120
                              ....|....|....|....|....|....|....|....|....|....|....|....|
```

```
KM359573.1 Bactrocera dorsalis      ------------------------------------------------------------
HQ446518.1 Bactrocera dorsalis      ------------------------------------------------------------
OQ626326.1 Bactrocera dorsalis      TCAGGAAAGAAGGAAACATTTGGATCTCTAGGAATAATTTATGCTATAATAGCAATTGGT
OP036560.1 Bactrocera dorsalis      TCAGGAAAGAAGGAAACATTTGGATCTCTAGGAATAATTTATGCTATAATAGCAATTGGT
OK175615.1 Bactrocera dorsalis      ------------------------------------------------------------
MW032650.1 Bactrocera dorsalis      ------------------------------------ATTTATGCTATAATGGCAATTGGT
MT257267.1 Bactrocera correcta      TCAGGAAAGAAGGAAACATTCGGATCTTTAGGAATAATTTATGCTATAATAGCAATTGGT
MK053681.1 Bactrocera correcta      -----------------------------------------------------TTCTGAA
KJ753908.1 Bactrocera correcta      ------------------------------------------------AAAGATATTGGA
MN016970.1 Bactrocera correcta      ------------------------------------------------AAAGATATTGGA
AY530905.1 Bactrocera correcta      ------------------------------------------------------------
JQ692631.1 Bactrocera correcta      -----------------------------------------------------------A
MT257819.1 Bactrocera zonata        TCAGGAAAGAAGGAAACATTTGGATCTCTAGGAATAATCTACGCTATAATAGCAATTGGT
MG881756.1 Bactrocera zonata        ---------------------------------------------CATAAAGATATTGGA
JX965418.1 Bactrocera zonata        -----------------------------------------------------------A
MT257315.1 Bactrocera zonata        TCAGGAAAGAAGGAAACATTTGGATCTCTAGGAATAATCTACGCTATAATAGCAATTGGT
MT258188.1 Bactrocera zonata        TCAGGAAAGAAGGAAACATTTGGATCTCTAGGAATAATCTACGCTATAATAGCAATTGGT
KJ142766.1 Zeugodacus cucurbit      TCAGGTAAAAAGGAAACATTTGGTTCCTTAGGTATAATTTACGCTATAATAGCAATTGGA
MN016983.1 Zeugodacus cucurbit      ---------------------------------------------CATAAAGATATTGGA
KP851001.1 Zeugodacus cucurbit      -----------------------------------------------------------A
OL701253.1 Zeugodacus cucurbit      ---------------------------------------------CATAAAGATATTGGA
OK103985.1 Zeugodacus cucurbit      ------------------------------------------------------------
MW300735.1 Zeugodacus cucurbit      ------------------------------------------------------------
KT175576.1 Zeugodacus tau           ---------------------------------------------------GATATTGGA
OL701270.1 Zeugodacus tau           ------------------------------------------------------------
MT257465.1 Zeugodacus tau           TCGGGTAAAAAGGAAACATTCGGATCCTTAGGTATAATTTATGCTATAATAGCAATTGGG
MK249718.1 Zeugodacus tau           -------------------------------------------ATCATAAAGATATTGGA
KJ753948.1 Bactrocera tau           ------------------------------------------------AAAGATATTGGA
MT456363.1 Bactrocera digressa      TCAGGGAAAAAGGAAACATTTGGTTCCCTAGGAATAATTTATGCTATAATAGCAATTGGT
MG683319.1 Bactrocera digressa      ------------------------------------------------------------
GQ458048.1 Bactrocera caudata       ------TTCCAATGCACTAATCTGCCATATTAGAAGTTAGTTAAAAGAGGTAGTTCAGAA
FJ903493.1 Bactrocera caudata       ------------------------------------------------------------
JX559676.1 Bactrocera caudata       -----------------------------------------------------------A
KF659996.1 Bactrocera caudata       -----------------------------------------------------------A
MK125511.1 Zeugodacus caudatus      ------------------------------------------------------------
AF423109.1 Bactrocera caudata       ------------------------------------------------------------
MK053684.1 Bactrocera nigrofem      -----------------------------------------------------TTCTGAA
MK660603.1 Bactrocera nigrofem      ------------------------------------------------------------
MH172171.1 Bactrocera nigrofem      ------------------------------------------------------------
KT594975.1 Bactrocera scutella      TCTGGTAAAAAAGAAACATTCGGTTCTTTAGGAATAATTTATGCAATAATAGCAATTGGA
MK053674.1 Zeugodacus scutella      -----------------------------------------------------TTCTGAA
KM024429.1 Bactrocera scutella      ------------------------------------------------AAAGATATGGGA
KT588387.1 Zeugodacus scutella      ------------------------------------------------------------
KF660074.1 Bactrocera scutella      -----------------------------------------------------------A
KJ753902.1 Bactrocera cilifera      ------------------------------------------------AAAGATATTGGA
MH395849.1 Zeugodacus cilifer      ---------------------------------------------------------GGA
JX266417.1 Bactrocera cilifera      -----------------------------------------------------------A
KF660011.1 Bactrocera cilifera      -----------------------------------------------------------A
MF095190.1 Zeugodacus cilifer      -----------------------------------------------------------A

                                             130       140       150       160       170       180
                                    ....|....|....|....|....|....|....|....|....|....|....|....|
KM359573.1 Bactrocera dorsalis      ------------------------------------------------------GTTGAT
HQ446518.1 Bactrocera dorsalis      ------------------------------------------------------------
```

```
OQ626326.1 Bactrocera dorsalis      CTATTAGGATTTATTGTATGAGCTCATCACATATTCACAGTAGGAA---TAGAT..A...
OP036560.1 Bactrocera dorsalis      CTATTAGGATTTATTGTATGAGCTCATCACATATTCACAGTAGGAA---TAGAT..A...
OK175615.1 Bactrocera dorsalis      ------------------------------------------------------------
MW032650.1 Bactrocera dorsalis      CTATTAGGATTTATTGTATGAGCTCATCACATATTCACAGTAGGAA---TAGAT..A...
MT257267.1 Bactrocera correcta      CTCTTAGGATTCATTGTATGAGCTCATCACATATTCACTGTAGGAA---TAGAT..A..C
MK053681.1 Bactrocera correcta      TAACTATGTTCAGCTGGGGGAGTATTTTGGAGTCATTCAATTGAAGAACTAAGTTG.ATA
KJ753908.1 Bactrocera correcta      ACCTTATATTTTATTTTCGGGGC---CTGAGCAGGAATAGTTGGGA---CATCCC..AGA
MN016970.1 Bactrocera correcta      ACCTTATATTTTATTTTCGGAGC---CTGAGCAGGAATAGTTGGGA---CATCCC..AGA
AY530905.1 Bactrocera correcta      -----------------------------------TACAGTTGGAA---TAGAC......
JQ692631.1 Bactrocera correcta      ACCTTATATTTTATTTTCGGGGC---CTGAGCAGGAATAGTTGGGA---CATCCC..AGA
MT257819.1 Bactrocera zonata        CTTCTAGGATTTATTGTATGAGCACATCACATATTCACAGTAGGAA---TAGAT..A..C
MG881756.1 Bactrocera zonata        ACCTTATATTTTATTTTCGGAGC---CTGAGCAGGTATAGTTGGAA---CATCTC..AGA
JX965418.1 Bactrocera zonata        ACCTTATATTTTATTTTCGGAGC---CTGAGCGGGGATAGTTGGAA---CATCCC..AGA
MT257315.1 Bactrocera zonata        CTTTTAGGATTTATTGTATGAGCCCATCACATATTCACAGTAGGAA---TAGAT..A..C
MT258188.1 Bactrocera zonata        CTTTTAGGGTTTATTGTATGAGCCCATCACATATTCACAGTAGGAA---TAGAT..A..C
KJ142766.1 Zeugodacus cucurbit      TTACTTGGATTTATTGTATGAGCCCACCATATATTTACAGTAGGTA---TAGAT......
MN016983.1 Zeugodacus cucurbit      ACATTATATTTTATTTTCGGAGC---TTGAGCAGGTATAGTGGGAA---CATCTC..AGA
KP851001.1 Zeugodacus cucurbit      ACATTATATTTTATTTTCGGAGC---TTGAGCAGGTATAGTGGGAA---CATCTC..AGA
OL701253.1 Zeugodacus cucurbit      ACATTATATTTTATTTTCGGAGC---TTGAGCAGGTATAGTGGGAA---CATCTC..AGA
OK103985.1 Zeugodacus cucurbit      ----------------------C---TTGAGCAGGTATAGTGGGAA---CATCTC..AGA
MW300735.1 Zeugodacus cucurbit      ------TATTTTATTTTCGGAGC---TTGAGCAGGTATAGTGGGAA---CATCTC..AGA
KT175576.1 Zeugodacus tau           ACATTATATTTTATTTTCGGAGC---TTGAGCAGGTATAGTAGGAA---CATCTC..AGA
OL701270.1 Zeugodacus tau           ------------------------------------------------------------
MT257465.1 Zeugodacus tau           TTACTTGGGTTTATTGTATGAGCTCATCATATATTTACAGTAGGTA---TAGAT......
MK249718.1 Zeugodacus tau           ACATTATATTTTATTTTCGGAGC---CTGAGCAGGCATAGTAGGAA---CATCCC..AGA
KJ753948.1 Bactrocera tau           ACATTATATTTTATTTTCGGAGC---TTGAGCAGGTATAGTAGGAA---CATCTC..AGA
MT456363.1 Bactrocera digressa      CTTTTAGGCTTCATTGTATGAGCTCACCACATATTTACAGTAGGAA---TAGAT..A..C
MG683319.1 Bactrocera digressa      -------------------------------------------------TACTTTGA.GA
GQ458048.1 Bactrocera caudata       TAACTGTGTTCTGCTGGAGGTGTGTTTTGAAGTCATTCAATTGAAGAACTAAGTTG.ATA
FJ903493.1 Bactrocera caudata       -----------------------------------TACAGTTGGAA---TAGAC......
JX559676.1 Bactrocera caudata       ACCTTATATTTTATCTTCGGTGC---TTGAGCAGGTATAGTAGGAA---CATCTT.GAGA
KF659996.1 Bactrocera caudata       ACCTTGTATTTTATCTTCGGTGC---TTGAGCAGGTATAGTAGGGA---CATCTT.AAGA
MK125511.1 Zeugodacus caudatus     ------------ATCTTCGGTGC---TTGAGCAGGTATAGTAGGAA---CATCTT.GAGA
AF423109.1 Bactrocera caudata       ------------------------------------------------------------
MK053684.1 Bactrocera nigrofem      TAACTATGTTCAGCTGGAGGAGTATTTTGTAGTCATTCAATTGAAGAACTAAGTTG.ATA
MK660603.1 Bactrocera nigrofem      ------------ATTTTCGGAGC---CTGAGCAGGGATAGTAGGAA---CATCTC..AGA
MH172171.1 Bactrocera nigrofem      ------------------------------------------------------------
KT594975.1 Bactrocera scutella      CTACTTGGTTTTATTGTTTGAGCTCATCATATATTTACTGTAGGTA---TAGAC..A..C
MK053674.1 Zeugodacus scutella      TAACTATGTTCGGCTGGTGGAGTATTTTGAAGTCATTCAATTGAAGAACTAAGTTG.ATA
KM024429.1 Bactrocera scutella      ACTTTATATTTTATCTTCGGTGC---TTGAGCAGGTATAGTAGGAA---CATCTT.AAGA
KT588387.1 Zeugodacus scutella      -------ATTTTATCTTGCGTGC---TTGAGCAGGTATAGTAGGAA---CATCTT.AAGA
KF660074.1 Bactrocera scutella      ACTTTATATTTTATCTTCGGTGC---TTGAGCAGGTATAGTAGGAA---CATCTT.AAGA
KJ753902.1 Bactrocera cilifera      ACTTTATACTTCATTTTCGGAGC---TTGAGCAGGTATAGTGGGTA---CATCTC..AGA
MH395849.1 Zeugodacus cilifer      ACTTTATACTTCATTTTCGGAGC---TTGAGCAGGTATAGTAGGTA---CATCTC..AGA
JX266417.1 Bactrocera cilifera      ACTTTATACTTCATTTTCGGAGC---TTGAGCAGGTATAGTGGGTA---CATCTC..AGA
KF660011.1 Bactrocera cilifera      ACTTTATACTTCATTTTCGGAGC---TTGAGCAGGTATAGTAGGTA---CATCTC..AGA
MF095190.1 Zeugodacus cilifer      ACTTTATACTTCATTTTCGGAGC---TTGAGCAGGTATAGTAGGTA---CATCTC..AGA

                                             190       200       210       220       230       240
                                    ....|....|....|....|....|....|....|....|....|....|....|....|
KM359573.1 Bactrocera dorsalis      ACTCGTGCCTATTTCACTTCAGCTA--CAATAATTATTGCG-GTACCCACAGGTATTAAA
HQ446518.1 Bactrocera dorsalis      -----------------------------------.AG...-..-.....G..G......
OQ626326.1 Bactrocera dorsalis      .........................--.............A-..................
OP036560.1 Bactrocera dorsalis      .........................--..............-..................
```

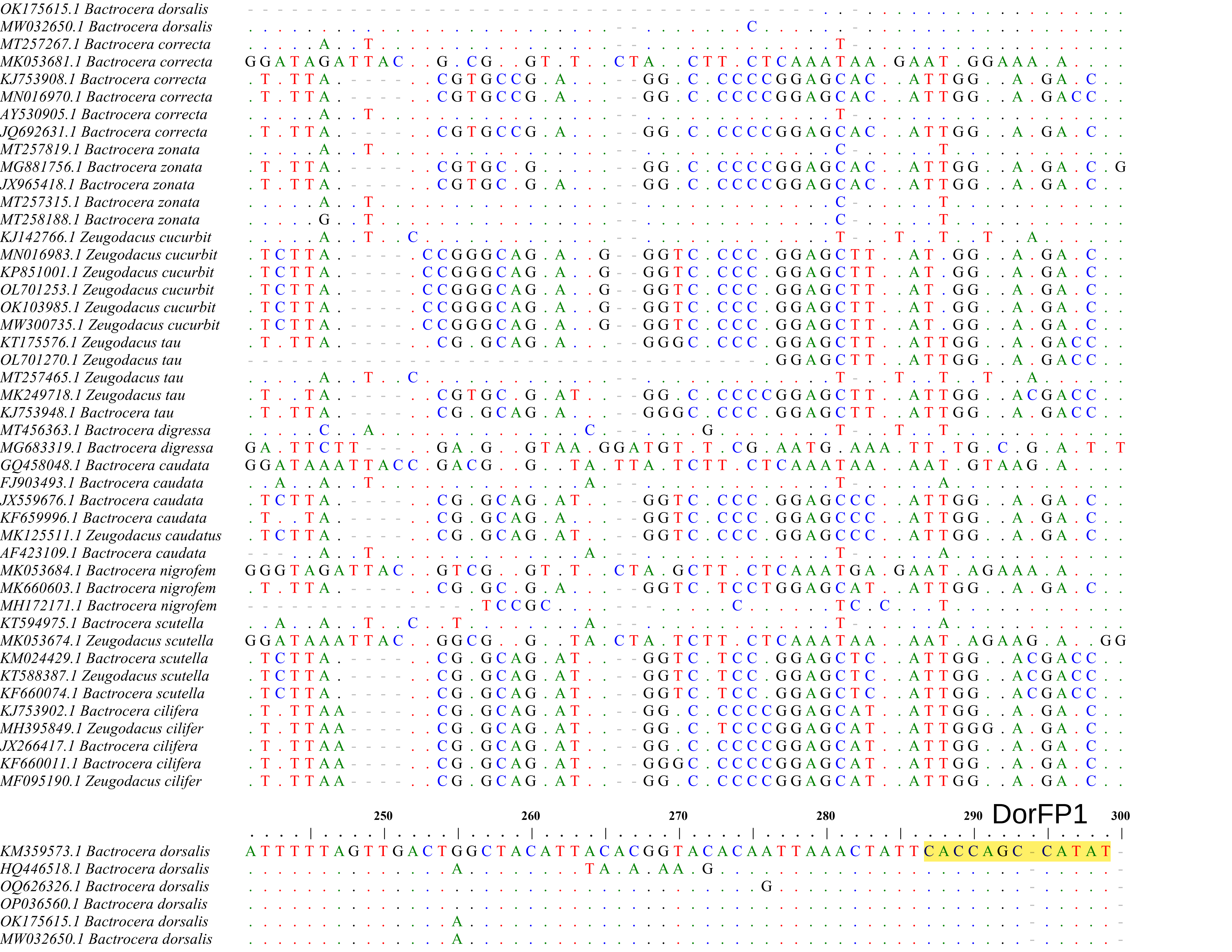

OK175615.1 Bactrocera dorsalis
MW032650.1 Bactrocera dorsalis
MT257267.1 Bactrocera correcta
MK053681.1 Bactrocera correcta
KJ753908.1 Bactrocera correcta
MN016970.1 Bactrocera correcta
AY530905.1 Bactrocera correcta
JQ692631.1 Bactrocera correcta
MT257819.1 Bactrocera zonata
MG881756.1 Bactrocera zonata
JX965418.1 Bactrocera zonata
MT257315.1 Bactrocera zonata
MT258188.1 Bactrocera zonata
KJ142766.1 Zeugodacus cucurbit
MN016983.1 Zeugodacus cucurbit
KP851001.1 Zeugodacus cucurbit
OL701253.1 Zeugodacus cucurbit
OK103985.1 Zeugodacus cucurbit
MW300735.1 Zeugodacus cucurbit
KT175576.1 Zeugodacus tau
OL701270.1 Zeugodacus tau
MT257465.1 Zeugodacus tau
MK249718.1 Zeugodacus tau
KJ753948.1 Bactrocera tau
MT456363.1 Bactrocera digressa
MG683319.1 Bactrocera digressa
GQ458048.1 Bactrocera caudata
FJ903493.1 Bactrocera caudata
JX559676.1 Bactrocera caudata
KF659996.1 Bactrocera caudata
MK125511.1 Zeugodacus caudatus
AF423109.1 Bactrocera caudata
MK053684.1 Bactrocera nigrofem
MK660603.1 Bactrocera nigrofem
MH172171.1 Bactrocera nigrofem
KT594975.1 Bactrocera scutella
MK053674.1 Zeugodacus scutella
KM024429.1 Bactrocera scutella
KT588387.1 Zeugodacus scutella
KF660074.1 Bactrocera scutella
KJ753902.1 Bactrocera cilifera
MH395849.1 Zeugodacus cilifer
JX266417.1 Bactrocera cilifera
KF660011.1 Bactrocera cilifera
MF095190.1 Zeugodacus cilifer
250
260
270
280
290
DorFP1
300
KM359573.1 Bactrocera dorsalis
ATTTTTAGTTGACTGGCTACATTACACGGTACACAATTAAACTATTCACCAGC-CATAT-
HQ446518.1 Bactrocera dorsalis
OQ626326.1 Bactrocera dorsalis
OP036560.1 Bactrocera dorsalis
OK175615.1 Bactrocera dorsalis
MW032650.1 Bactrocera dorsalis

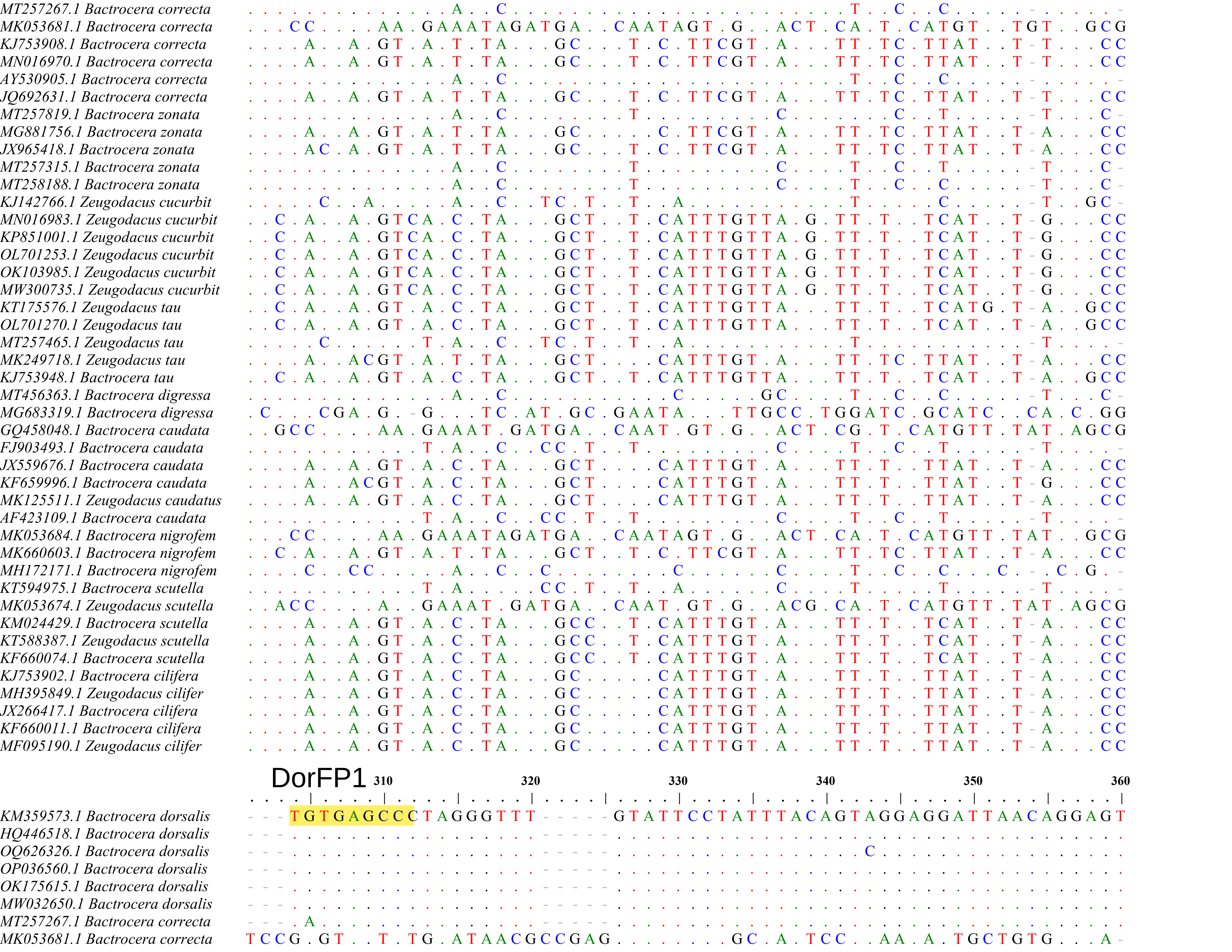
MT257267.1 Bactrocera correcta
MK053681.1 Bactrocera correcta
KJ753908.1 Bactrocera correcta
MN016970.1 Bactrocera correcta
AY530905.1 Bactrocera correcta
JQ692631.1 Bactrocera correcta
MT257819.1 Bactrocera zonata
MG881756.1 Bactrocera zonata
JX965418.1 Bactrocera zonata
MT257315.1 Bactrocera zonata
MT258188.1 Bactrocera zonata
KJ142766.1 Zeugodacus cucurbit
MN016983.1 Zeugodacus cucurbit
KP851001.1 Zeugodacus cucurbit
OL701253.1 Zeugodacus cucurbit
OK103985.1 Zeugodacus cucurbit
MW300735.1 Zeugodacus cucurbit
KT175576.1 Zeugodacus tau
OL701270.1 Zeugodacus tau
MT257465.1 Zeugodacus tau
MK249718.1 Zeugodacus tau
KJ753948.1 Bactrocera tau
MT456363.1 Bactrocera digressa
MG683319.1 Bactrocera digressa
GQ458048.1 Bactrocera caudata
FJ903493.1 Bactrocera caudata
JX559676.1 Bactrocera caudata
KF659996.1 Bactrocera caudata
MK125511.1 Zeugodacus caudatus
AF423109.1 Bactrocera caudata
MK053684.1 Bactrocera nigrofem
MK660603.1 Bactrocera nigrofem
MH172171.1 Bactrocera nigrofem
KT594975.1 Bactrocera scutella
MK053674.1 Zeugodacus scutella
KM024429.1 Bactrocera scutella
KT588387.1 Zeugodacus scutella
KF660074.1 Bactrocera scutella
KJ753902.1 Bactrocera cilifera
MH395849.1 Zeugodacus cilifer
JX266417.1 Bactrocera cilifera
KF660011.1 Bactrocera cilifera
MF095190.1 Zeugodacus cilifer
DorFP1
310
320
330
340
350
360
KM359573.1 Bactrocera dorsalis
TGTGAGCCCTAGGGTTT GTATTCCTATTTACAGTAGGAGGATTAACAGGAGT
HQ446518.1 Bactrocera dorsalis
OQ626326.1 Bactrocera dorsalis
OP036560.1 Bactrocera dorsalis
OK175615.1 Bactrocera dorsalis
MW032650.1 Bactrocera dorsalis
MT257267.1 Bactrocera correcta
MK053681.1 Bactrocera correcta

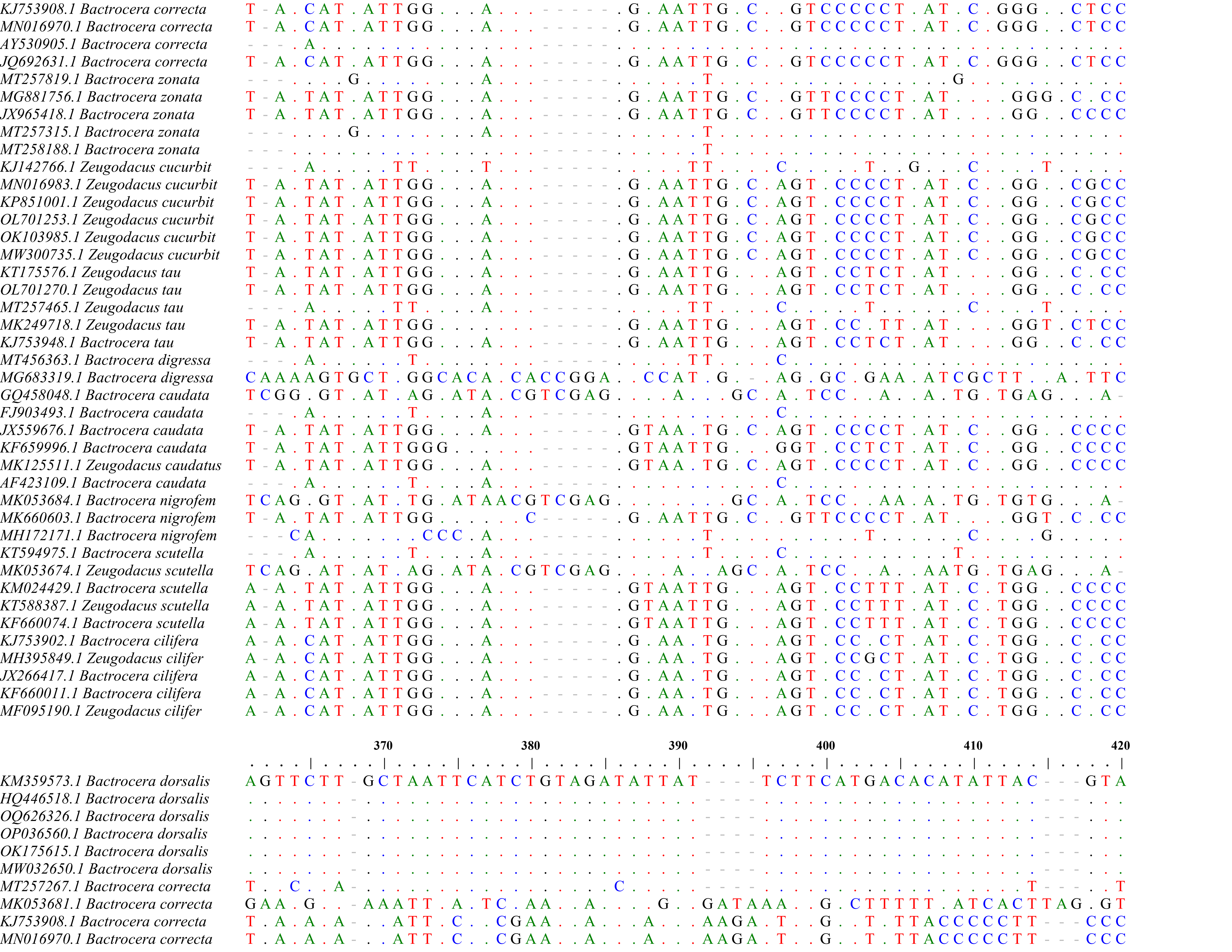
KJ753908.1 Bactrocera correcta
MN016970.1 Bactrocera correcta
AY530905.1 Bactrocera correcta
JQ692631.1 Bactrocera correcta
MT257819.1 Bactrocera zonata
MG881756.1 Bactrocera zonata
JX965418.1 Bactrocera zonata
MT257315.1 Bactrocera zonata
MT258188.1 Bactrocera zonata
KJ142766.1 Zeugodacus cucurbit
MN016983.1 Zeugodacus cucurbit
KP851001.1 Zeugodacus cucurbit
OL701253.1 Zeugodacus cucurbit
OK103985.1 Zeugodacus cucurbit
MW300735.1 Zeugodacus cucurbit
KT175576.1 Zeugodacus tau
OL701270.1 Zeugodacus tau
MT257465.1 Zeugodacus tau
MK249718.1 Zeugodacus tau
KJ753948.1 Bactrocera tau
MT456363.1 Bactrocera digressa
MG683319.1 Bactrocera digressa
GQ458048.1 Bactrocera caudata
FJ903493.1 Bactrocera caudata
JX559676.1 Bactrocera caudata
KF659996.1 Bactrocera caudata
MK125511.1 Zeugodacus caudatus
AF423109.1 Bactrocera caudata
MK053684.1 Bactrocera nigrofem
MK660603.1 Bactrocera nigrofem
MH172171.1 Bactrocera nigrofem
KT594975.1 Bactrocera scutella
MK053674.1 Zeugodacus scutella
KM024429.1 Bactrocera scutella
KT588387.1 Zeugodacus scutella
KF660074.1 Bactrocera scutella
KJ753902.1 Bactrocera cilifera
MH395849.1 Zeugodacus cilifer
JX266417.1 Bactrocera cilifera
KF660011.1 Bactrocera cilifera
MF095190.1 Zeugodacus cilifer
370
380
390
400
410
420
KM359573.1 Bactrocera dorsalis
HQ446518.1 Bactrocera dorsalis
OQ626326.1 Bactrocera dorsalis
OP036560.1 Bactrocera dorsalis
OK175615.1 Bactrocera dorsalis
MW032650.1 Bactrocera dorsalis
MT257267.1 Bactrocera correcta
MK053681.1 Bactrocera correcta
KJ753908.1 Bactrocera correcta
MN016970.1 Bactrocera correcta

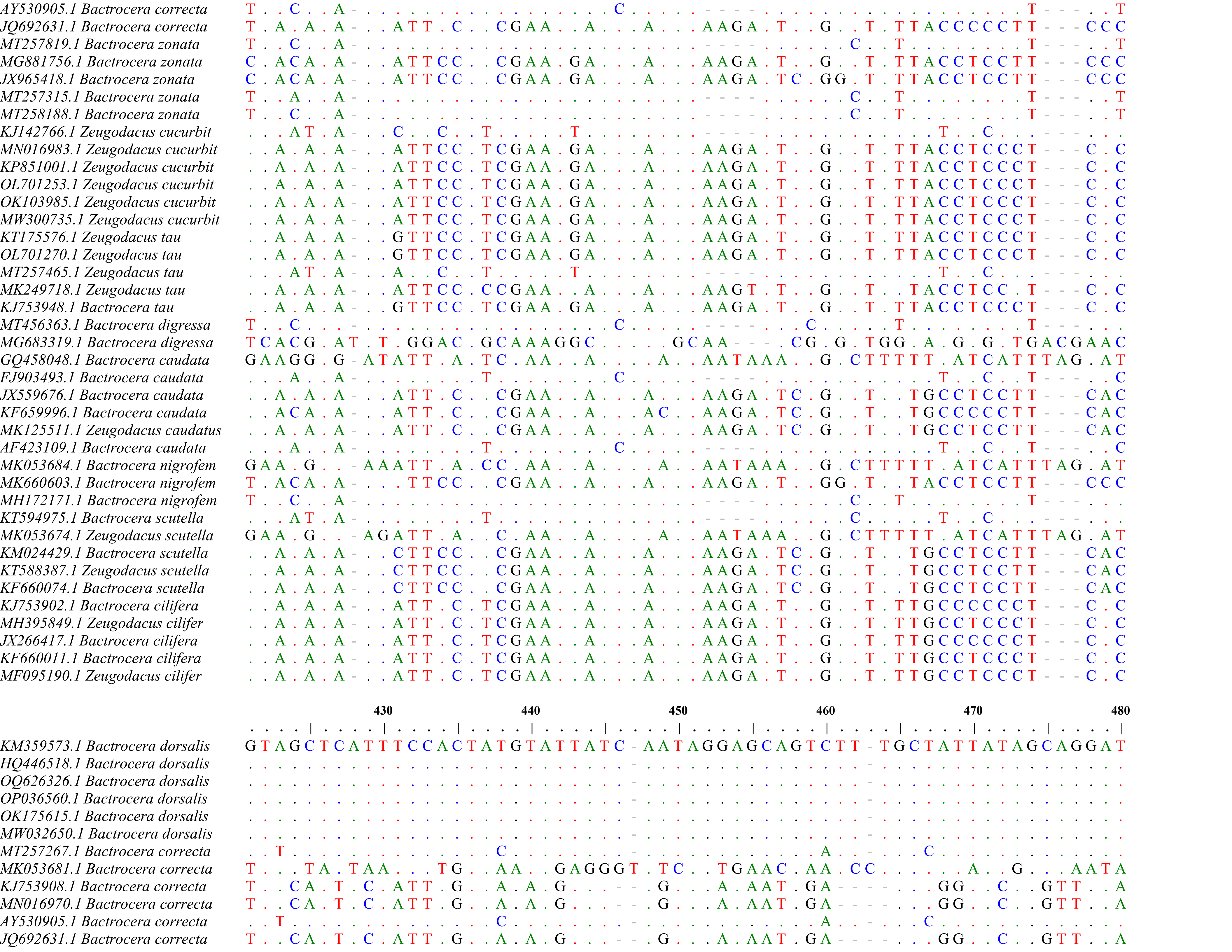
AY530905.1 Bactrocera correcta
JQ692631.1 Bactrocera correcta
MT257819.1 Bactrocera zonata
MG881756.1 Bactrocera zonata
JX965418.1 Bactrocera zonata
MT257315.1 Bactrocera zonata
MT258188.1 Bactrocera zonata
KJ142766.1 Zeugodacus cucurbit
MN016983.1 Zeugodacus cucurbit
KP851001.1 Zeugodacus cucurbit
OL701253.1 Zeugodacus cucurbit
OK103985.1 Zeugodacus cucurbit
MW300735.1 Zeugodacus cucurbit
KT175576.1 Zeugodacus tau
OL701270.1 Zeugodacus tau
MT257465.1 Zeugodacus tau
MK249718.1 Zeugodacus tau
KJ753948.1 Bactrocera tau
MT456363.1 Bactrocera digressa
MG683319.1 Bactrocera digressa
GQ458048.1 Bactrocera caudata
FJ903493.1 Bactrocera caudata
JX559676.1 Bactrocera caudata
KF659996.1 Bactrocera caudata
MK125511.1 Zeugodacus caudatus
AF423109.1 Bactrocera caudata
MK053684.1 Bactrocera nigrofem
MK660603.1 Bactrocera nigrofem
MH172171.1 Bactrocera nigrofem
KT594975.1 Bactrocera scutella
MK053674.1 Zeugodacus scutella
KM024429.1 Bactrocera scutella
KT588387.1 Zeugodacus scutella
KF660074.1 Bactrocera scutella
KJ753902.1 Bactrocera cilifera
MH395849.1 Zeugodacus cilifer
JX266417.1 Bactrocera cilifera
KF660011.1 Bactrocera cilifera
MF095190.1 Zeugodacus cilifer
430
440
450
460
470
480
KM359573.1 Bactrocera dorsalis
HQ446518.1 Bactrocera dorsalis
OQ626326.1 Bactrocera dorsalis
OP036560.1 Bactrocera dorsalis
OK175615.1 Bactrocera dorsalis
MW032650.1 Bactrocera dorsalis
MT257267.1 Bactrocera correcta
MK053681.1 Bactrocera correcta
KJ753908.1 Bactrocera correcta
MN016970.1 Bactrocera correcta
AY530905.1 Bactrocera correcta
JQ692631.1 Bactrocera correcta

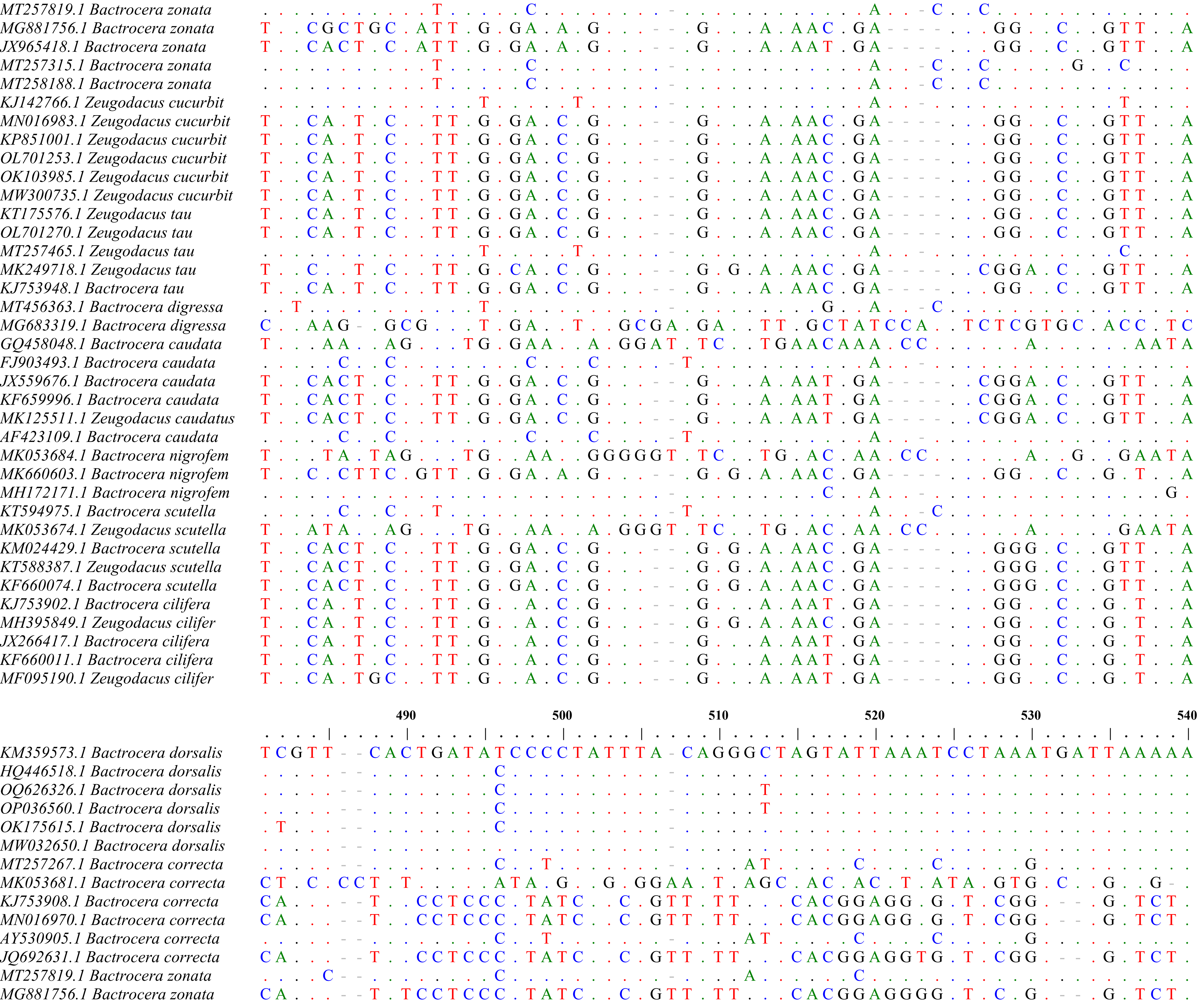
MT257819.1 Bactrocera zonata
MG881756.1 Bactrocera zonata
JX965418.1 Bactrocera zonata
MT257315.1 Bactrocera zonata
MT258188.1 Bactrocera zonata
KJ142766.1 Zeugodacus cucurbit
MN016983.1 Zeugodacus cucurbit
KP851001.1 Zeugodacus cucurbit
OL701253.1 Zeugodacus cucurbit
OK103985.1 Zeugodacus cucurbit
MW300735.1 Zeugodacus cucurbit
KT175576.1 Zeugodacus tau
OL701270.1 Zeugodacus tau
MT257465.1 Zeugodacus tau
MK249718.1 Zeugodacus tau
KJ753948.1 Bactrocera tau
MT456363.1 Bactrocera digressa
MG683319.1 Bactrocera digressa
GQ458048.1 Bactrocera caudata
FJ903493.1 Bactrocera caudata
JX559676.1 Bactrocera caudata
KF659996.1 Bactrocera caudata
MK125511.1 Zeugodacus caudatus
AF423109.1 Bactrocera caudata
MK053684.1 Bactrocera nigrofem
MK660603.1 Bactrocera nigrofem
MH172171.1 Bactrocera nigrofem
KT594975.1 Bactrocera scutella
MK053674.1 Zeugodacus scutella
KM024429.1 Bactrocera scutella
KT588387.1 Zeugodacus scutella
KF660074.1 Bactrocera scutella
KJ753902.1 Bactrocera cilifera
MH395849.1 Zeugodacus cilifer
JX266417.1 Bactrocera cilifera
KF660011.1 Bactrocera cilifera
MF095190.1 Zeugodacus cilifer
490
500
510
520
530
540
KM359573.1 Bactrocera dorsalis
HQ446518.1 Bactrocera dorsalis
OQ626326.1 Bactrocera dorsalis
OP036560.1 Bactrocera dorsalis
OK175615.1 Bactrocera dorsalis
MW032650.1 Bactrocera dorsalis
MT257267.1 Bactrocera correcta
MK053681.1 Bactrocera correcta
KJ753908.1 Bactrocera correcta
MN016970.1 Bactrocera correcta
AY530905.1 Bactrocera correcta
JQ692631.1 Bactrocera correcta
MT257819.1 Bactrocera zonata
MG881756.1 Bactrocera zonata

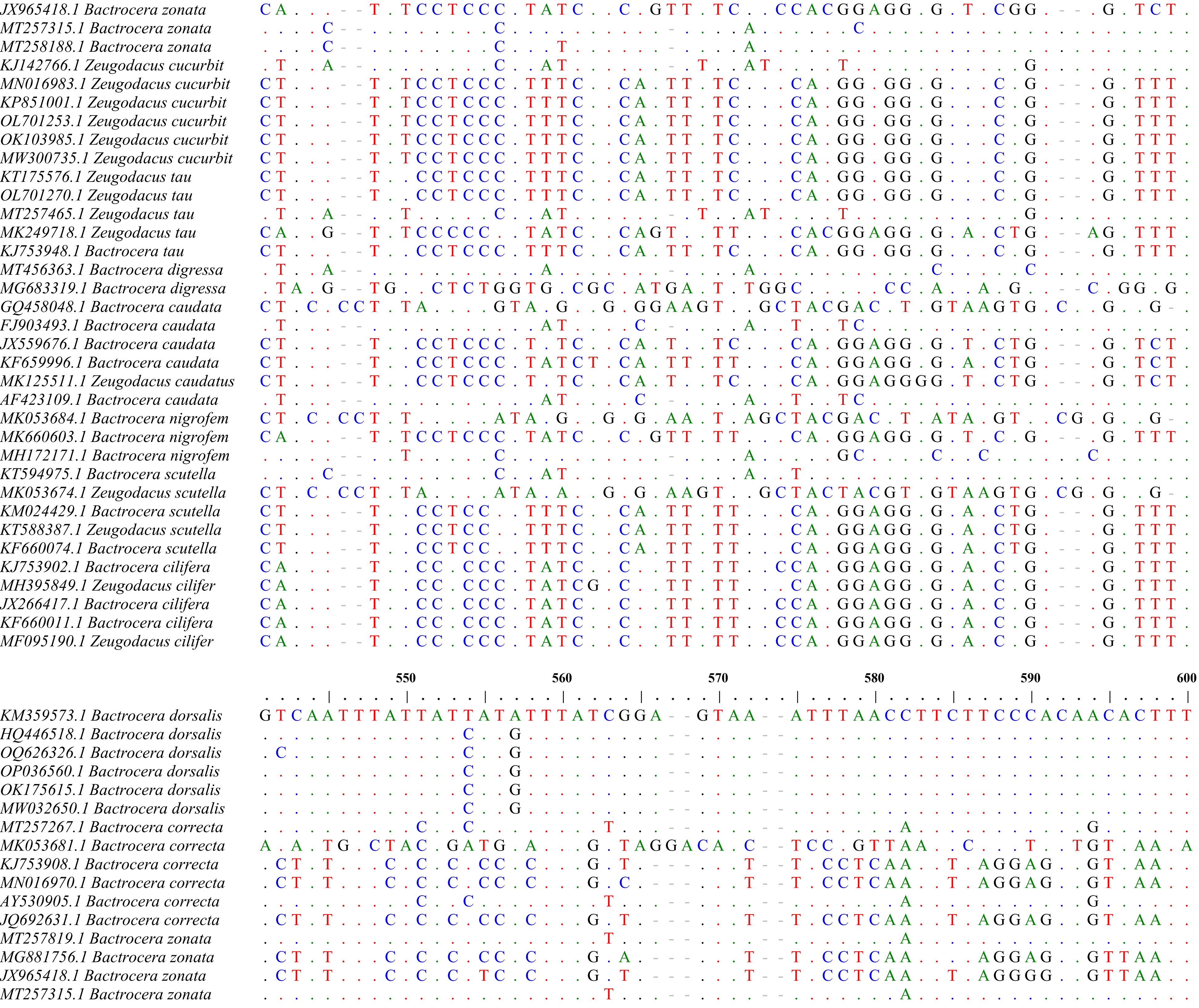

JX965418.1 Bactrocera zonata
MT257315.1 Bactrocera zonata
MT258188.1 Bactrocera zonata
KJ142766.1 Zeugodacus cucurbit
MN016983.1 Zeugodacus cucurbit
KP851001.1 Zeugodacus cucurbit
OL701253.1 Zeugodacus cucurbit
OK103985.1 Zeugodacus cucurbit
MW300735.1 Zeugodacus cucurbit
KT175576.1 Zeugodacus tau
OL701270.1 Zeugodacus tau
MT257465.1 Zeugodacus tau
MK249718.1 Zeugodacus tau
KJ753948.1 Bactrocera tau
MT456363.1 Bactrocera digressa
MG683319.1 Bactrocera digressa
GQ458048.1 Bactrocera caudata
FJ903493.1 Bactrocera caudata
JX559676.1 Bactrocera caudata
KF659996.1 Bactrocera caudata
MK125511.1 Zeugodacus caudatus
AF423109.1 Bactrocera caudata
MK053684.1 Bactrocera nigrofem
MK660603.1 Bactrocera nigrofem
MH172171.1 Bactrocera nigrofem
KT594975.1 Bactrocera scutella
MK053674.1 Zeugodacus scutella
KM024429.1 Bactrocera scutella
KT588387.1 Zeugodacus scutella
KF660074.1 Bactrocera scutella
KJ753902.1 Bactrocera cilifera
MH395849.1 Zeugodacus cilifer
JX266417.1 Bactrocera cilifera
KF660011.1 Bactrocera cilifera
MF095190.1 Zeugodacus cilifer
550
560
570
580
590
600
KM359573.1 Bactrocera dorsalis
HQ446518.1 Bactrocera dorsalis
OQ626326.1 Bactrocera dorsalis
OP036560.1 Bactrocera dorsalis
OK175615.1 Bactrocera dorsalis
MW032650.1 Bactrocera dorsalis
MT257267.1 Bactrocera correcta
MK053681.1 Bactrocera correcta
KJ753908.1 Bactrocera correcta
MN016970.1 Bactrocera correcta
AY530905.1 Bactrocera correcta
JQ692631.1 Bactrocera correcta
MT257819.1 Bactrocera zonata
MG881756.1 Bactrocera zonata
JX965418.1 Bactrocera zonata
MT257315.1 Bactrocera zonata

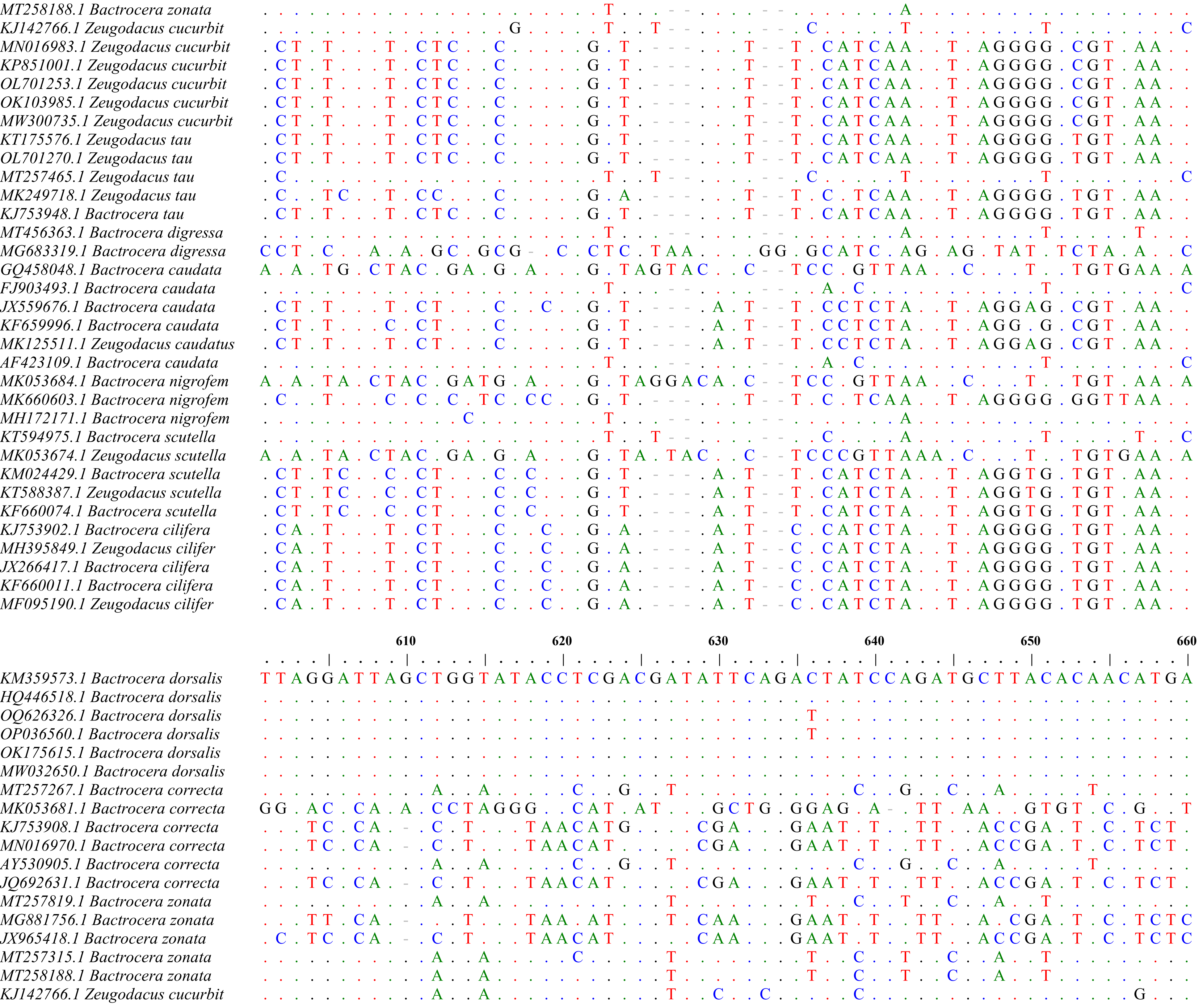
MT258188.1 Bactrocera zonata
KJ142766.1 Zeugodacus cucurbit
MN016983.1 Zeugodacus cucurbit
KP851001.1 Zeugodacus cucurbit
OL701253.1 Zeugodacus cucurbit
OK103985.1 Zeugodacus cucurbit
MW300735.1 Zeugodacus cucurbit
KT175576.1 Zeugodacus tau
OL701270.1 Zeugodacus tau
MT257465.1 Zeugodacus tau
MK249718.1 Zeugodacus tau
KJ753948.1 Bactrocera tau
MT456363.1 Bactrocera digressa
MG683319.1 Bactrocera digressa
GQ458048.1 Bactrocera caudata
FJ903493.1 Bactrocera caudata
JX559676.1 Bactrocera caudata
KF659996.1 Bactrocera caudata
MK125511.1 Zeugodacus caudatus
AF423109.1 Bactrocera caudata
MK053684.1 Bactrocera nigrofem
MK660603.1 Bactrocera nigrofem
MH172171.1 Bactrocera nigrofem
KT594975.1 Bactrocera scutella
MK053674.1 Zeugodacus scutella
KM024429.1 Bactrocera scutella
KT588387.1 Zeugodacus scutella
KF660074.1 Bactrocera scutella
KJ753902.1 Bactrocera cilifera
MH395849.1 Zeugodacus cilifer
JX266417.1 Bactrocera cilifera
KF660011.1 Bactrocera cilifera
MF095190.1 Zeugodacus cilifer
610
620
630
640
650
660
KM359573.1 Bactrocera dorsalis
HQ446518.1 Bactrocera dorsalis
OQ626326.1 Bactrocera dorsalis
OP036560.1 Bactrocera dorsalis
OK175615.1 Bactrocera dorsalis
MW032650.1 Bactrocera dorsalis
MT257267.1 Bactrocera correcta
MK053681.1 Bactrocera correcta
KJ753908.1 Bactrocera correcta
MN016970.1 Bactrocera correcta
AY530905.1 Bactrocera correcta
JQ692631.1 Bactrocera correcta
MT257819.1 Bactrocera zonata
MG881756.1 Bactrocera zonata
JX965418.1 Bactrocera zonata
MT257315.1 Bactrocera zonata
MT258188.1 Bactrocera zonata
KJ142766.1 Zeugodacus cucurbit

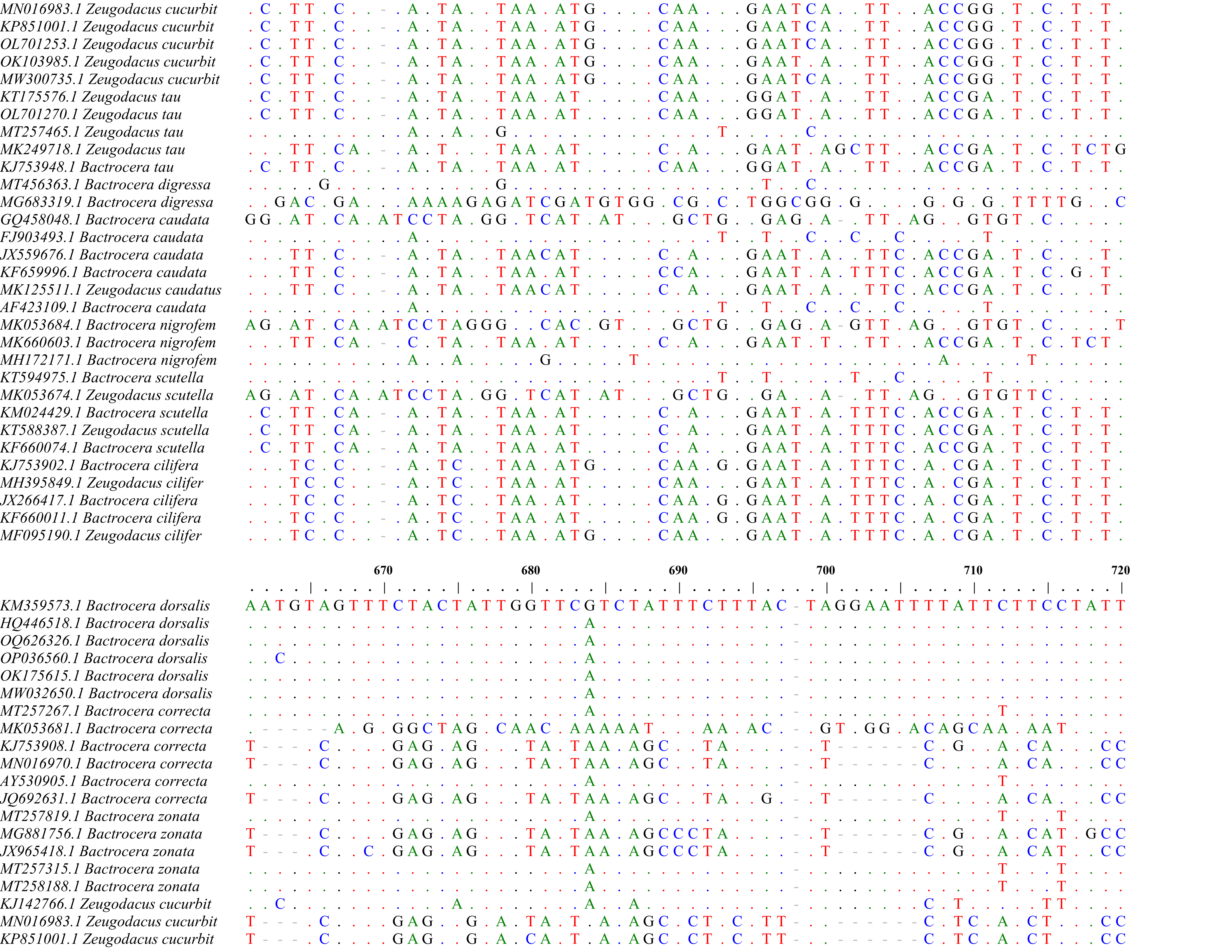
MN016983.1 Zeugodacus cucurbit
KP851001.1 Zeugodacus cucurbit
OL701253.1 Zeugodacus cucurbit
OK103985.1 Zeugodacus cucurbit
MW300735.1 Zeugodacus cucurbit
KT175576.1 Zeugodacus tau
OL701270.1 Zeugodacus tau
MT257465.1 Zeugodacus tau
MK249718.1 Zeugodacus tau
KJ753948.1 Bactrocera tau
MT456363.1 Bactrocera digressa
MG683319.1 Bactrocera digressa
GQ458048.1 Bactrocera caudata
FJ903493.1 Bactrocera caudata
JX559676.1 Bactrocera caudata
KF659996.1 Bactrocera caudata
MK125511.1 Zeugodacus caudatus
AF423109.1 Bactrocera caudata
MK053684.1 Bactrocera nigrofem
MK660603.1 Bactrocera nigrofem
MH172171.1 Bactrocera nigrofem
KT594975.1 Bactrocera scutella
MK053674.1 Zeugodacus scutella
KM024429.1 Bactrocera scutella
KT588387.1 Zeugodacus scutella
KF660074.1 Bactrocera scutella
KJ753902.1 Bactrocera cilifera
MH395849.1 Zeugodacus cilifer
JX266417.1 Bactrocera cilifera
KF660011.1 Bactrocera cilifera
MF095190.1 Zeugodacus cilifer
670
680
690
700
710
720
KM359573.1 Bactrocera dorsalis
AATGTAGTTTCTACTATTGGTTCGTCTATTTCTTTAC-TAGGAATTTTATTCTTCCTATT
HQ446518.1 Bactrocera dorsalis
OQ626326.1 Bactrocera dorsalis
OP036560.1 Bactrocera dorsalis
OK175615.1 Bactrocera dorsalis
MW032650.1 Bactrocera dorsalis
MT257267.1 Bactrocera correcta
MK053681.1 Bactrocera correcta
KJ753908.1 Bactrocera correcta
MN016970.1 Bactrocera correcta
AY530905.1 Bactrocera correcta
JQ692631.1 Bactrocera correcta
MT257819.1 Bactrocera zonata
MG881756.1 Bactrocera zonata
JX965418.1 Bactrocera zonata
MT257315.1 Bactrocera zonata
MT258188.1 Bactrocera zonata
KJ142766.1 Zeugodacus cucurbit
MN016983.1 Zeugodacus cucurbit
KP851001.1 Zeugodacus cucurbit

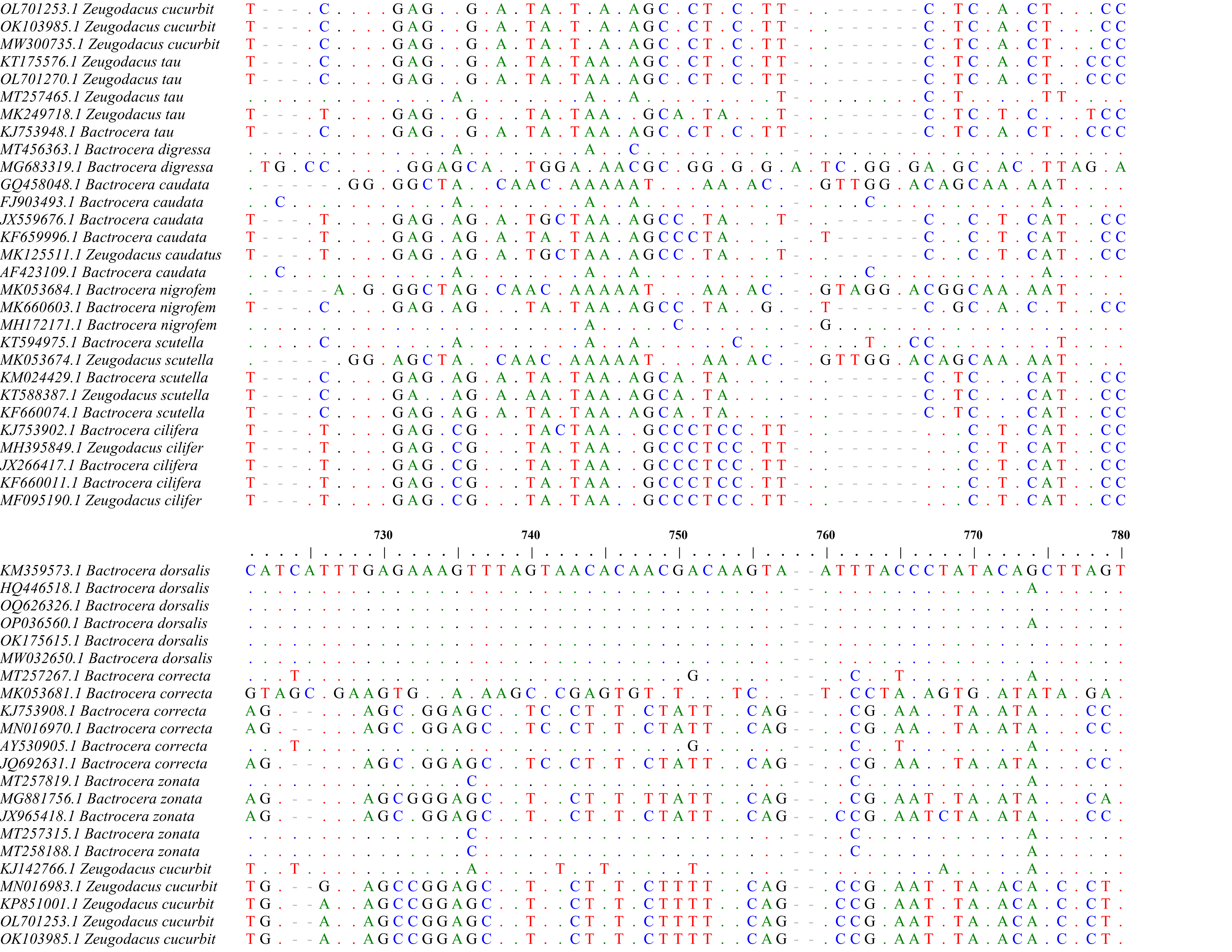

OL701253.1 Zeugodacus cucurbit
OK103985.1 Zeugodacus cucurbit
MW300735.1 Zeugodacus cucurbit
KT175576.1 Zeugodacus tau
OL701270.1 Zeugodacus tau
MT257465.1 Zeugodacus tau
MK249718.1 Zeugodacus tau
KJ753948.1 Bactrocera tau
MT456363.1 Bactrocera digressa
MG683319.1 Bactrocera digressa
GQ458048.1 Bactrocera caudata
FJ903493.1 Bactrocera caudata
JX559676.1 Bactrocera caudata
KF659996.1 Bactrocera caudata
MK125511.1 Zeugodacus caudatus
AF423109.1 Bactrocera caudata
MK053684.1 Bactrocera nigrofem
MK660603.1 Bactrocera nigrofem
MH172171.1 Bactrocera nigrofem
KT594975.1 Bactrocera scutella
MK053674.1 Zeugodacus scutella
KM024429.1 Bactrocera scutella
KT588387.1 Zeugodacus scutella
KF660074.1 Bactrocera scutella
KJ753902.1 Bactrocera cilifera
MH395849.1 Zeugodacus cilifer
JX266417.1 Bactrocera cilifera
KF660011.1 Bactrocera cilifera
MF095190.1 Zeugodacus cilifer
730 740 750 760 770 780
KM359573.1 Bactrocera dorsalis
HQ446518.1 Bactrocera dorsalis
OQ626326.1 Bactrocera dorsalis
OP036560.1 Bactrocera dorsalis
OK175615.1 Bactrocera dorsalis
MW032650.1 Bactrocera dorsalis
MT257267.1 Bactrocera correcta
MK053681.1 Bactrocera correcta
KJ753908.1 Bactrocera correcta
MN016970.1 Bactrocera correcta
AY530905.1 Bactrocera correcta
JQ692631.1 Bactrocera correcta
MT257819.1 Bactrocera zonata
MG881756.1 Bactrocera zonata
JX965418.1 Bactrocera zonata
MT257315.1 Bactrocera zonata
MT258188.1 Bactrocera zonata
KJ142766.1 Zeugodacus cucurbit
MN016983.1 Zeugodacus cucurbit
KP851001.1 Zeugodacus cucurbit
OL701253.1 Zeugodacus cucurbit
OK103985.1 Zeugodacus cucurbit

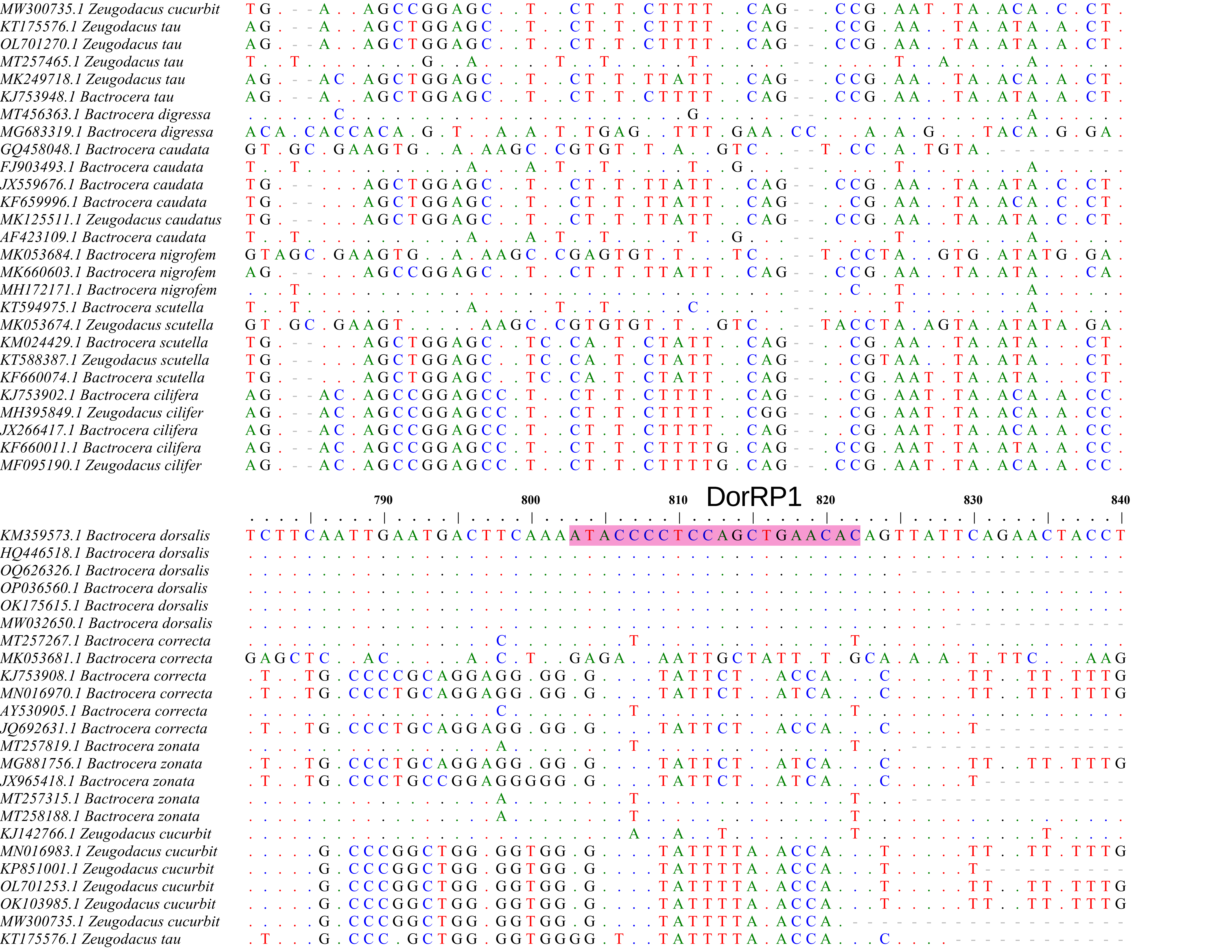

MW300735.1 Zeugodacus cucurbit
KT175576.1 Zeugodacus tau
OL701270.1 Zeugodacus tau
MT257465.1 Zeugodacus tau
MK249718.1 Zeugodacus tau
KJ753948.1 Bactrocera tau
MT456363.1 Bactrocera digressa
MG683319.1 Bactrocera digressa
GQ458048.1 Bactrocera caudata
FJ903493.1 Bactrocera caudata
JX559676.1 Bactrocera caudata
KF659996.1 Bactrocera caudata
MK125511.1 Zeugodacus caudatus
AF423109.1 Bactrocera caudata
MK053684.1 Bactrocera nigrofem
MK660603.1 Bactrocera nigrofem
MH172171.1 Bactrocera nigrofem
KT594975.1 Bactrocera scutella
MK053674.1 Zeugodacus scutella
KM024429.1 Bactrocera scutella
KT588387.1 Zeugodacus scutella
KF660074.1 Bactrocera scutella
KJ753902.1 Bactrocera cilifera
MH395849.1 Zeugodacus cilifer
JX266417.1 Bactrocera cilifera
KF660011.1 Bactrocera cilifera
MF095190.1 Zeugodacus cilifer
790
800
810
DorRP1
820
830
840
KM359573.1 Bactrocera dorsalis
HQ446518.1 Bactrocera dorsalis
OQ626326.1 Bactrocera dorsalis
OP036560.1 Bactrocera dorsalis
OK175615.1 Bactrocera dorsalis
MW032650.1 Bactrocera dorsalis
MT257267.1 Bactrocera correcta
MK053681.1 Bactrocera correcta
KJ753908.1 Bactrocera correcta
MN016970.1 Bactrocera correcta
AY530905.1 Bactrocera correcta
JQ692631.1 Bactrocera correcta
MT257819.1 Bactrocera zonata
MG881756.1 Bactrocera zonata
JX965418.1 Bactrocera zonata
MT257315.1 Bactrocera zonata
MT258188.1 Bactrocera zonata
KJ142766.1 Zeugodacus cucurbit
MN016983.1 Zeugodacus cucurbit
KP851001.1 Zeugodacus cucurbit
OL701253.1 Zeugodacus cucurbit
OK103985.1 Zeugodacus cucurbit
MW300735.1 Zeugodacus cucurbit
KT175576.1 Zeugodacus tau

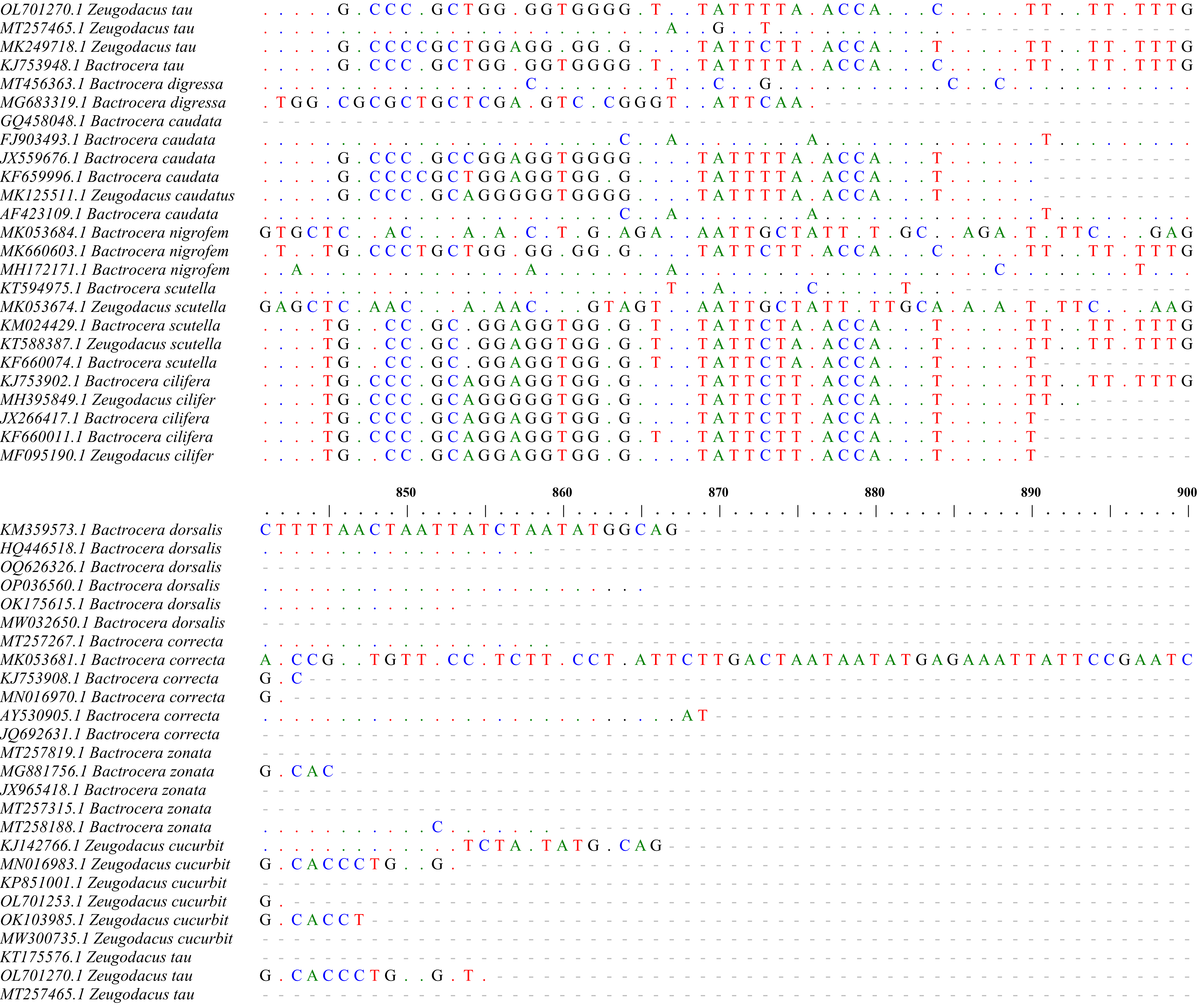

OL701270.1 Zeugodacus tau
MT257465.1 Zeugodacus tau
MK249718.1 Zeugodacus tau
KJ753948.1 Bactrocera tau
MT456363.1 Bactrocera digressa
MG683319.1 Bactrocera digressa
GQ458048.1 Bactrocera caudata
FJ903493.1 Bactrocera caudata
JX559676.1 Bactrocera caudata
KF659996.1 Bactrocera caudata
MK125511.1 Zeugodacus caudatus
AF423109.1 Bactrocera caudata
MK053684.1 Bactrocera nigrofem
MK660603.1 Bactrocera nigrofem
MH172171.1 Bactrocera nigrofem
KT594975.1 Bactrocera scutella
MK053674.1 Zeugodacus scutella
KM024429.1 Bactrocera scutella
KT588387.1 Zeugodacus scutella
KF660074.1 Bactrocera scutella
KJ753902.1 Bactrocera cilifera
MH395849.1 Zeugodacus cilifer
JX266417.1 Bactrocera cilifera
KF660011.1 Bactrocera cilifera
MF095190.1 Zeugodacus cilifer
850
860
870
880
890
900
KM359573.1 Bactrocera dorsalis
HQ446518.1 Bactrocera dorsalis
OQ626326.1 Bactrocera dorsalis
OP036560.1 Bactrocera dorsalis
OK175615.1 Bactrocera dorsalis
MW032650.1 Bactrocera dorsalis
MT257267.1 Bactrocera correcta
MK053681.1 Bactrocera correcta
KJ753908.1 Bactrocera correcta
MN016970.1 Bactrocera correcta
AY530905.1 Bactrocera correcta
JQ692631.1 Bactrocera correcta
MT257819.1 Bactrocera zonata
MG881756.1 Bactrocera zonata
JX965418.1 Bactrocera zonata
MT257315.1 Bactrocera zonata
MT258188.1 Bactrocera zonata
KJ142766.1 Zeugodacus cucurbit
MN016983.1 Zeugodacus cucurbit
KP851001.1 Zeugodacus cucurbit
OL701253.1 Zeugodacus cucurbit
OK103985.1 Zeugodacus cucurbit
MW300735.1 Zeugodacus cucurbit
KT175576.1 Zeugodacus tau
OL701270.1 Zeugodacus tau
MT257465.1 Zeugodacus tau

```
MK249718.1 Zeugodacus tau        G . C A C C C T G . . G - - - - - - - - - - - - - - - - - - - - - - - - - - - - - - - - - - - - - - - - - - - - - - - -
KJ753948.1 Bactrocera tau        G . C - - - - - - - - - - - - - - - - - - - - - - - - - - - - - - - - - - - - - - - - - - - - - - - - - - - - - - - - -
MT456363.1 Bactrocera digressa   . . . . . . . . . . . C . - - - - - - - - - - - - - - - - - - - - - - - - - - - - - - - - - - - - - - - - - - - - - -
MG683319.1 Bactrocera digressa   - - - - - - - - - - - - - - - - - - - - - - - - - - - - - - - - - - - - - - - - - - - - - - - - - - - - - - - - - - - -
GQ458048.1 Bactrocera caudata    - - - - - - - - - - - - - - - - - - - - - - - - - - - - - - - - - - - - - - - - - - - - - - - - - - - - - - - - - - - -
FJ903493.1 Bactrocera caudata    . . . . . . . . . . . C . T C T A . T A T G . C A G A T T A G T G C A T T - - - - - - - - - - - - - - - - - - - - - - -
JX559676.1 Bactrocera caudata    - - - - - - - - - - - - - - - - - - - - - - - - - - - - - - - - - - - - - - - - - - - - - - - - - - - - - - - - - - - -
KF659996.1 Bactrocera caudata    - - - - - - - - - - - - - - - - - - - - - - - - - - - - - - - - - - - - - - - - - - - - - - - - - - - - - - - - - - - -
MK125511.1 Zeugodacus caudatus   - - - - - - - - - - - - - - - - - - - - - - - - - - - - - - - - - - - - - - - - - - - - - - - - - - - - - - - - - - - -
AF423109.1 Bactrocera caudata    . . . . . . . . . . . C . T C - - - - - - - - - - - - - - - - - - - - - - - - - - - - - - - - - - - - - - - - - - - - -
MK053684.1 Bactrocera nigrofem   A . C C A . . T G T T . C C . T C T T . C C T . A T T C T T G A C T A A T A A T A T G G G A A A T T A T T C C G A A T C
MK660603.1 Bactrocera nigrofem   G . C A C C - - - - - - - - - - - - - - - - - - - - - - - - - - - - - - - - - - - - - - - - - - - - - - - - - - - - - -
MH172171.1 Bactrocera nigrofem   . . . . . . . . . . . C . . . . . . . . . . . . . . . A T T A G T G C A T T G G A C C C - - - - - - - - - - - - - - - -
KT594975.1 Bactrocera scutella   - - - - - - - - - - - - - - - - - - - - - - - - - - - - - - - - - - - - - - - - - - - - - - - - - - - - - - - - - - - -
MK053674.1 Zeugodacus scutella   A A C C G . . T G T T . C C . T . T T A C C A . A T T C T T G G C T A A T A A T A T G G G A G A T T A T T C C G A A T C
KM024429.1 Bactrocera scutella   G . C - - - - - - - - - - - - - - - - - - - - - - - - - - - - - - - - - - - - - - - - - - - - - - - - - - - - - - - - -
KT588387.1 Zeugodacus scutella   G . C A C C C - - - - - - - - - - - - - - - - - - - - - - - - - - - - - - - - - - - - - - - - - - - - - - - - - - - - -
KF660074.1 Bactrocera scutella   - - - - - - - - - - - - - - - - - - - - - - - - - - - - - - - - - - - - - - - - - - - - - - - - - - - - - - - - - - - -
KJ753902.1 Bactrocera cilifera   G . C - - - - - - - - - - - - - - - - - - - - - - - - - - - - - - - - - - - - - - - - - - - - - - - - - - - - - - - - -
MH395849.1 Zeugodacus cilifer    - - - - - - - - - - - - - - - - - - - - - - - - - - - - - - - - - - - - - - - - - - - - - - - - - - - - - - - - - - - -
JX266417.1 Bactrocera cilifera   - - - - - - - - - - - - - - - - - - - - - - - - - - - - - - - - - - - - - - - - - - - - - - - - - - - - - - - - - - - -
KF660011.1 Bactrocera cilifera   - - - - - - - - - - - - - - - - - - - - - - - - - - - - - - - - - - - - - - - - - - - - - - - - - - - - - - - - - - - -
MF095190.1 Zeugodacus cilifer    - - - - - - - - - - - - - - - - - - - - - - - - - - - - - - - - - - - - - - - - - - - - - - - - - - - - - - - - - - - -
```

```
                                 . . . .
KM359573.1 Bactrocera dorsalis   - - - -
HQ446518.1 Bactrocera dorsalis   - - - -
OQ626326.1 Bactrocera dorsalis   - - - -
OP036560.1 Bactrocera dorsalis   - - - -
OK175615.1 Bactrocera dorsalis   - - - -
MW032650.1 Bactrocera dorsalis   - - - -
MT257267.1 Bactrocera correcta   - - - -
MK053681.1 Bactrocera correcta   C T G G
KJ753908.1 Bactrocera correcta   - - - -
MN016970.1 Bactrocera correcta   - - - -
AY530905.1 Bactrocera correcta   - - - -
JQ692631.1 Bactrocera correcta   - - - -
MT257819.1 Bactrocera zonata     - - - -
MG881756.1 Bactrocera zonata     - - - -
JX965418.1 Bactrocera zonata     - - - -
MT257315.1 Bactrocera zonata     - - - -
MT258188.1 Bactrocera zonata     - - - -
KJ142766.1 Zeugodacus cucurbit   - - - -
MN016983.1 Zeugodacus cucurbit   - - - -
KP851001.1 Zeugodacus cucurbit   - - - -
OL701253.1 Zeugodacus cucurbit   - - - -
OK103985.1 Zeugodacus cucurbit   - - - -
MW300735.1 Zeugodacus cucurbit   - - - -
KT175576.1 Zeugodacus tau        - - - -
OL701270.1 Zeugodacus tau        - - - -
MT257465.1 Zeugodacus tau        - - - -
MK249718.1 Zeugodacus tau        - - - -
KJ753948.1 Bactrocera tau        - - - -
```

MT456363.1 Bactrocera digressa	-	-	-	-
MG683319.1 Bactrocera digressa	-	-	-	-
GQ458048.1 Bactrocera caudata	-	-	-	-
FJ903493.1 Bactrocera caudata	-	-	-	-
JX559676.1 Bactrocera caudata	-	-	-	-
KF659996.1 Bactrocera caudata	-	-	-	-
MK125511.1 Zeugodacus caudatus	-	-	-	-
AF423109.1 Bactrocera caudata	-	-	-	-
MK053684.1 Bactrocera nigrofem	C	T	G	G
MK660603.1 Bactrocera nigrofem	-	-	-	-
MH172171.1 Bactrocera nigrofem	-	-	-	-
KT594975.1 Bactrocera scutella	-	-	-	-
MK053674.1 Zeugodacus scutella	C	T	G	G
KM024429.1 Bactrocera scutella	-	-	-	-
KT588387.1 Zeugodacus scutella	-	-	-	-
KF660074.1 Bactrocera scutella	-	-	-	-
KJ753902.1 Bactrocera cilifera	-	-	-	-
MH395849.1 Zeugodacus cilifer	-	-	-	-
JX266417.1 Bactrocera cilifera	-	-	-	-
KF660011.1 Bactrocera cilifera	-	-	-	-
MF095190.1 Zeugodacus cilifer	-	-	-	-
