# Supplementary material for: A simple PCR-based quick detection of the economically important oriental fruit fly, Bactrocera dorsalis (Hendel) from India
Source: Front Plant Sci. 2024 Jul 9;15:1399718. doi: 10.3389/fpls.2024.1399718 (PMC11263087; doi:10.3389/fpls.2024.1399718)
Supplement: Supplementary file 4 [file Table_1.docx]

Supplementary Material

# Supplementary Figures and Tables

## Supplementary Figures

**Supplementary Figure 1** Distribution map of *Bactrocera dorsalis* (Hendel)*.* Accessed on 09/03/2024; Source: <https://gd.eppo.int/taxon/DACUDO/distribution>, *B. dorsalis* having transient pest status in Australia, Italy (France and California, USA).

**Supplementary Figure 2** Photographs of the different immature stages of *B. dorsalis*, taken under a Zoomstar-III, Dewinter stereo-zoom microscope mounted with a Sony IMX586 (48 MP) camera. **(A)** eggs, **(B)** maggots and **(C)** pupae.

**Supplementary Figure 3** The multiple sequence alignment of mtCOI gene sequences from fruit flies, utilized in the experimental study as detailed in Table S1, highlights the position of DorFP1 and DorRP1 SSPs in yellow and pink, respectively.

## Supplementary Tables

**Supplementary Table 1** Details of the different fruit fly samples used in this study.

| **Fruit fly species** | **NCBI Accession numbers of the sequences used in the study** | |
| --- | --- | --- |
|  | **Mitochondrial cytochrome oxidase-1 gene region (mtCOI)** | **Complete mitochondrial genome** |
| *B. dorsalis* | KM359573.1, HQ446518.1, OQ626326.1, OP036560.1, OK175615.1, MW032650.1 | PP493825.1, PP493824.1, PP493823.1, PP493817.1 |
| *B. zonata* | MT257819.1, MG881756.1, JX965418.1, MK564020.1, MT258188.1 | KP296150.1, NC_027725.1, MN583036.1, MT121277.1 |
| *B. correcta* | MT257267.1, MK053681.1, KJ753908.1, MN016970.1, AY530905.1, JQ692631.1 | JX456552.1, NC_018787.1, MT121259.1, |
| *B. digressa* | KU985297.1, OK559998, MT456363.1, MG683319.1 | - |
| *B. nigrofemoralis* | MK053684.1, MK660603.1, MH172171.1 | - |
| *Z. cucurbitae* | KJ142766.1, MN016983.1, KP851001.1, OL701253.1, OK103985.1, MW300735.1 | MT121249.1, JN635562.1, NC_016056.1, OQ158899.1 |
| *Z. tau* | KT175576.1, OL701270.1, MT257465.1, MK249718.1, KJ753948.1 | MF966383.1, MT121257.1, MH900081.1, KP711431.1 |
| *Z. caudatus* | GQ458048.1, JX559676.1, KF659996.1, MK125511.1, AF423109.1, JN542417.1 | KT625492.2, KT625491.2, MT180970.1, NC_062801.1 |
| *Z. scutellaris* | KT594975.1, MK053674.1, KM024429.1, KT588387.1, KF660074.1 | MZ233625.1, NC_061658.1, MT121255.1, MH900080.1 |
| *Z. cilifera* | KJ753902.1, MH395849.1, JX266417.1, KF660011.1, MF095190.1 | - |
| *Carpomya vesuviana* | - | MT121230.1 |

***B:*** *Bactrocera* & ***Z:*** *Zeugodacus*

**
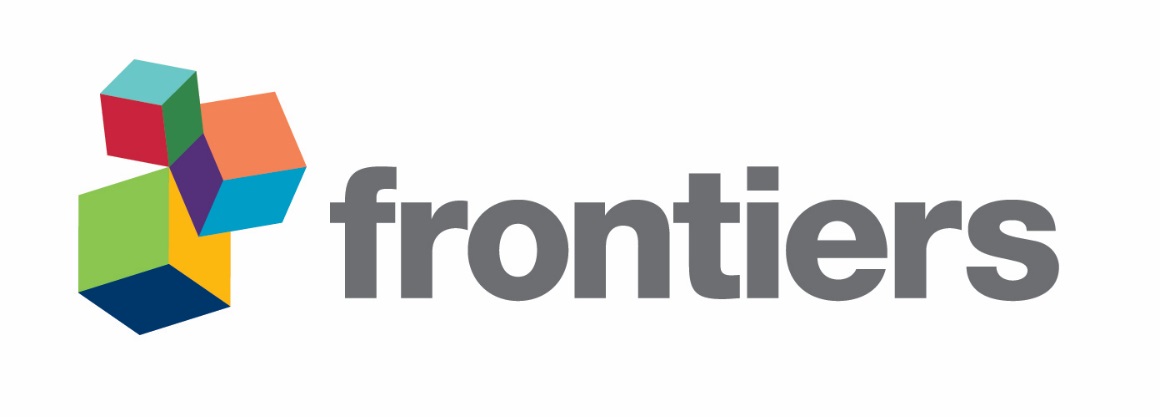
**
